# Molecular evaluation of five different isolation methods for extracellular vesicles reveals different clinical applicability and subcellular origin

**DOI:** 10.1002/jev2.12128

**Published:** 2021-07-22

**Authors:** Rosanne E. Veerman, Loes Teeuwen, Paulo Czarnewski, Gözde Güclüler Akpinar, AnnSofi Sandberg, Xiaofang Cao, Maria Pernemalm, Lukas M. Orre, Susanne Gabrielsson, Maria Eldh

**Affiliations:** ^1^ Department of Clinical Immunology and Transfusion Medicine and Division of Immunology and Allergy, Department of Medicine Solna Karolinska University Hospital and Karolinska Institutet Stockholm Sweden; ^2^ Department of Oncology and Pathology Karolinska Institutet Science for Life Laboratory Solna Sweden; ^3^ Science for Life Laboratory Department of Biochemistry and Biophysics National Bioinformatics Infrastructure Sweden Stockholm University Solna Sweden

**Keywords:** EV isolation, EVs, exosomes, extracellular vesicles, microvesicles, MV, plasma, proteomics, subpopulations

## Abstract

Extracellular vesicles (EVs) are increasingly tested as therapeutic vehicles and biomarkers, but still EV subtypes are not fully characterised. To isolate EVs with few co‐isolated entities, a combination of methods is needed. However, this is time‐consuming and requires large sample volumes, often not feasible in most clinical studies or in studies where small sample volumes are available. Therefore, we compared EVs rendered by five commonly used methods based on different principles from conditioned cell medium and 250 μl or 3 ml plasma, that is, precipitation (ExoQuick ULTRA), membrane affinity (exoEasy Maxi Kit), size‐exclusion chromatography (qEVoriginal), iodixanol gradient (OptiPrep), and phosphatidylserine affinity (MagCapture). EVs were characterised by electron microscopy, Nanoparticle Tracking Analysis, Bioanalyzer, flow cytometry, and LC‐MS/MS. The different methods yielded samples of different morphology, particle size, and proteomic profile. For the conditioned medium, Izon 35 isolated the highest number of EV proteins followed by exoEasy, which also isolated fewer non‐EV proteins. For the plasma samples, exoEasy isolated a high number of EV proteins and few non‐EV proteins, while Izon 70 isolated the most EV proteins. We conclude that no method is perfect for all studies, rather, different methods are suited depending on sample type and interest in EV subtype, in addition to sample volume and budget.

## 1 BACKGROUND

Extracellular vesicles (EVs) are a diverse group of membrane‐enclosed vesicles of different cellular origin and size. EVs derived from cells undergoing apoptosis are called apoptotic bodies (50–1000 nm in diameter). EVs released from living cells include microvesicles (MVs), which bud off from the plasma membrane (100–1000 nm), and exosomes (30–150 nm) which are of endosomal origin and are released in the extracellular environment upon fusion of multivesicular bodies (MVBs) with the plasma membrane (Colombo et al., 2014; Théry et al., 2009; Yáñez‐Mó et al., 2015). However, several studies have also divided EVs into different subtypes based on size, density, content, and topology (Kowal et al., 2016; Lázaro‐Ibáñez et al., 2019), so this categorisation is likely not final.

EVs are released by all cells and found in all human body fluids such as urine, breast milk, and plasma (Admyre et al., 2007; Caby et al., 2005; Lässer et al., 2011). Their composition is made up of lipids, proteins, and nucleic acids, which differ depending on their cellular origin and state, and can be transferred to recipient cells (Lobb et al., 2017; Mathieu et al., 2019; Valadi et al., 2007). EVs play important roles in both immune activation and tolerance, and are involved in many diseases such as cancer, making them both attractive targets and vehicles in therapy (Karlsson et al., 2001; Markov et al., 2019; Pascucci et al., 2014; Peinado et al., 2012; Raposo et al., 1996; Zitvogel et al., 1998). In addition, EVs released during disease can carry disease specific markers making them of great interest and consequently they have an emerging role as biomarkers (Boukouris & Mathivanan, 2015; Danzer et al., 2012; Eldh et al., 2014; Hurwitz et al., 2016; Logozzi et al., 2009; Schou et al., 2020; Skog et al., 2008).

**FIGURE 1:**
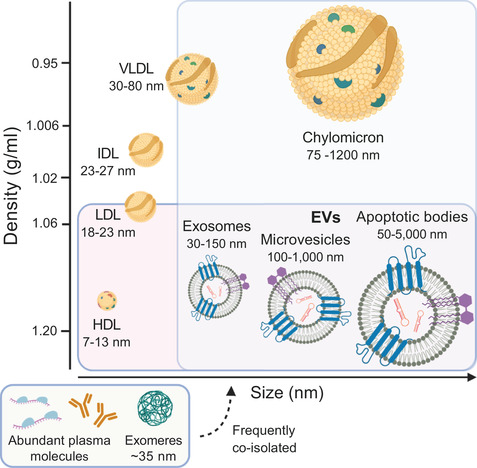
The complexity of extracellular vesicles and particles in plasma. Schematic overview of the distribution of lipoproteins and extracellular vesicles (EVs) according to size (nm) and density (g/ml). High‐density lipoprotein (HDL) (Rosenson et al., 2011), low‐density lipoprotein (LDL), intermediate‐density lipoprotein (IDL) (Wojczynski et al., 2011), very low‐density lipoprotein (VLDL) and chylomicrons (Feingold & Grunfeld, 2000) have overlapping sizes and densities with EVs. Furthermore, plasma contains many abundant proteins such as albumin, (immuno‐)globulins and fibrinogen. Moreover, coding and non‐coding RNAs are largely present in plasma, which are often protected from degradation by bound proteins (Yao et al., 2020). Also exomeres, small non‐membranous nanoparticles, are present in plasma (Zhang et al., 2018). Created with BioRender.com

Plasma‐derived EVs are especially interesting as biomarkers, since it can be taken as a liquid biopsy, a fast and relatively non‐invasive sampling method. However, plasma is a very complex body fluid consisting of soluble proteins such as albumin and multiple lipoproteins such as HDL, LDL, IDL, VLDL, and chylomicrons. Lipoproteins share several overlapping characteristics with EVs, for example, density, size, and lipid content (Figure 1) (Menard et al., 2018; Sódar et al., 2016; Yuana et al., 2014), and they dominate the plasma with an estimated 10^7^–10^9^‐fold excess of lipoprotein particles over EVs (Simonsen, 2017). This increases the difficulty to distinguish or fully separate lipoproteins from EVs (Karimi et al., 2018; Onódi et al., 2018; Sódar et al., 2016; Takov et al., 2019). When the intention is to use EVs as biomarkers it is important to, in most cases, isolate EVs from a small amount of plasma with little co‐enrichments of other plasma molecules, which is one of the challenges that is faced in EV research (Simonsen, 2017). Previously, sequential ultracentrifugation, or density gradient ultracentrifugation for a preparation with less co‐isolated entities, have been the most used methods for isolating EVs. However, nowadays several other methods such as size exclusion chromatography are widely used (Ayala‐Mar et al., 2019; Boriachek et al., 2018; Doyle & Wang, 2019).

One of the largest obstacles the field is currently facing is the unclear division of subtypes of EVs, and knowledge of how different isolation methods affect both the EV composition and the co‐isolated entities. There is a vast variety of different methods of isolating EVs in the literature, based on multiple EV characteristics. All these methods have both advantages and disadvantages, but first and foremost they all isolate a different mix of the EV subpopulations (Doyle & Wang, 2019). Thus, a better understanding of how the vesicle isolation procedure affects the yield and subpopulations of both EVs and co‐isolated entities is needed, which will also help when comparing the results of different published studies. Furthermore, it is also important to determine the definition of a “pure” EV sample. According to some studies, a pure EV preparation is defined by high particles‐to‐protein ratio (Deville et al., 2021; Webber & Clayton, 2013). However, this might be an oversimplification since a sample consisting of mainly lipoproteins would be considered as a pure EV sample based on the high particle number. To address this in an as unbiased and comprehensive way as possible, we have taken in different analyses such as transmission electron microscopy (TEM), Nanoparticle Tracking Analysis (NTA), flow cytometry, protein yield, RNA yield, and detailed proteomics (LC‐MS/MS) to characterize the isolated samples.

There are studies showing that, to achieve an increase in purity of the EV population, that is, fewer lipoproteins and other co‐isolated entities, a combination of several methods is required, particularly when isolating from plasma (Brennan et al., 2020; Karimi et al., 2018; Muller et al., 2014; Nordin et al., 2015; Onódi et al., 2018; Tulkens et al., 2020; Vaswani et al., 2017; Zhang et al., 2020). However, this makes EV isolation very time consuming and often requires a large starting volume. Therefore, combining several methods is inapplicable when it comes to studies using small sample volumes; for example, supernatant from tissue cultures and plasma derived from mice or patients and for most clinical studies. In large studies, there is a need for isolation methods that are time‐efficient and give a high yield of EVs. There is a large number of papers published which compare different commonly used methods for isolating EVs (Brennan et al., 2020; Buschmann et al., 2018; Buschmann et al., 2019; Deville et al., 2021; Helwa et al., 2017; Jung et al., 2020; Karimi et al., 2018; Onódi et al., 2018; Serrano‐Pertierra et al., 2019; Stranska et al., 2018; Takov et al., 2019; Tang et al., 2017; Tian et al., 2020; Van Deun et al., 2014). In some of these papers a large volume of plasma is used, which is as mentioned above, not feasible in most clinical studies. Furthermore, most of these studies compare methods from one sample type, while it is unknown whether some methods might be appropriate for plasma, but not for cell conditioned medium, or vice versa. With the aim to aid both in interpretation of the literature and in the selection of methods for future studies, we set out to compare five and four principally different methods of isolating EVs from conditioned cell medium and human plasma, respectively. To our knowledge, this is the first study that compares several different EV isolation methods in addition to different sample types and volumes side by side.

**FIGURE 2:**
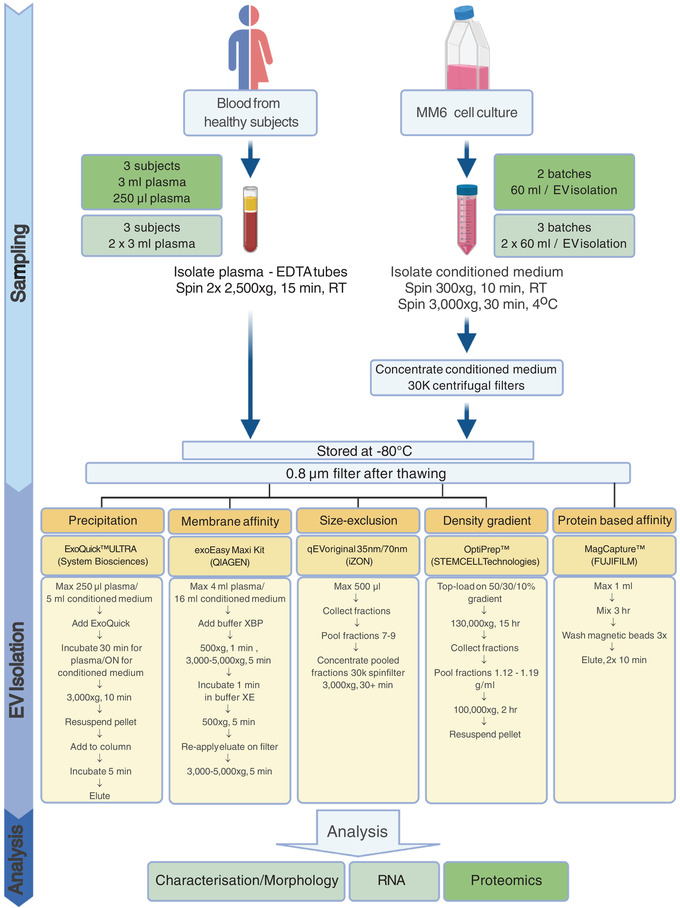
Schematic overview of the study design. Extracellular vesicle (EV) isolation and analysis from human plasma and MM6 cell culture using principally different commonly used EV isolation methods. Blood was collected in EDTA tubes from healthy subjects and centrifuged to produce platelet free plasma. Cells were cultured for 72 h in EV‐depleted fetal bovine serum prior to harvesting conditioned medium. Samples were frozen, thawed, filtered and subjected to four (plasma) and five (medium) EV isolation methods. Plasma was not isolated by MagCapture due to EDTA incompatibility. EV isolates were analysed by transmission electron microscopy, Nanoparticle Tracking Analysis, bead‐based flow cytometry, Bioanalyzer and LC‐MS/MS. Created with BioRender.com

EVs were isolated by precipitation (ExoQuick ULTRA), membrane affinity (exoEasy Maxi Kit), size‐exclusion chromatography (qEVoriginal 35 nm/70 nm), iodixanol gradient (OptiPrep), and phosphatidylserine affinity (MagCapture). EV purity, EV yield, feasibility, and cost was evaluated by comparing one volume of conditioned medium derived from the human monocytic cell line Mono‐Mac‐6 (MM6) and two different human plasma volumes. This was achieved by characterisation of the EVs by TEM, NTA, Bioanalyzer, flow cytometry, and LC‐MS/MS (study outline in Figure 2).

## 2 METHODS

We have submitted all relevant data of our experiments to the EV‐TRACK knowledgebase (EV‐TRACK ID: EV210020) (Van Deun et al., 2017).

## 3 CELL CULTURE

The non‐adherent human monocytic cell line Mono‐Mac‐6 (MM6), tested negative for mycoplasma, was cultured in RPMI‐1640 containing 10% heat inactivated Fetal Bovine Serum (FBS), 200 IU/ml penicillin, 200 μg/ml streptomycin, 1 mM sodium pyruvate, 1 × non‐essential amino acids, 1 mM L‐glutamine, O5003‐OPI (Oxaloacetate, Pyruvate, Insulin) supplement at 37°C in 5% CO_2_ with a seeding density of 2.5 × 10^5^ cells/ml. Cells were cultured in EV‐depleted medium for 72 h prior to conditioned medium isolation. EV‐depleted medium was prepared by overnight ultracentrifugation of heat inactivated FBS diluted in RPMI‐1640 (30%) 100,000 × g_avg_ at 4°C (k‐factor 211, Ti45 rotor, Beckman Coulter). After centrifugation, the 30% FBS solution was carefully poured off and filtered through a 0.22 μm filter. For serum free EV samples, cells were cultured in medium as described above but without FBS, 48 h prior to conditioned medium isolation. Conditioned medium was isolated from cells that were in passage 9–14 and had a viability of 90–96%. The cells were spun down (300 × g, 10 min at RT) and discarded, subsequently the cellular debris and apoptotic bodies were removed by 3000 × g centrifugation for 30 min at 4°C. The supernatant was concentrated (3000 × g, 30 min at 4°C) using Amicon Ultra 30K centrifugal filters (Merck). Concentrated supernatant, referred to as conditioned medium, was aliquoted in ∼ 1 ml, original volume 60 ml supernatant, and stored in cryo tubes at ‐80°C until further use.

## 4 PLASMA COLLECTION

Peripheral blood was collected from non‐fasting healthy donors in the morning, 1–3 h after breakfast (two females and one male, age 25–50). In brief, an average of 62 ml of blood was collected into K2E EDTA tubes and platelet‐free plasma (PFP) was prepared by 2 × centrifugation at 2500 × g for 15 min at RT. PFP was aliquoted and stored in cryo tubes at ‐80°C until further analysis (maximum of 16 months). All methods were carried out in accordance with relevant guidelines and regulations and all experimental protocols were approved by the Regional Ethical Review Board in Stockholm (2010/624‐31/4), all individuals were healthy (self‐reported), above 18 years of age and gave written and oral informed consent.

## 5 ISOLATION METHODS

Samples were defrosted at RT and filtered through 0.8 μm filters. From both conditioned cell medium and plasma, EV isolation was achieved by the following isolation methods: precipitation (ExoQuick ULTRA, System Biosciences), membrane affinity (exoEasy Maxi Kit, QIAGEN), size‐exclusion chromatography (qEVoriginal 35 nm and 70 nm, Izon) and iodixanol gradient (OptiPrep, STEMCELL Technologies). For conditioned cell medium also protein affinity for phosphatidylserine (PS), (MagCapture Exosome Isolation Kit PS, FUJIFILM) was used (Figure 2). MagCapture was excluded for plasma as this method is not compatible with EDTA without first performing an additional buffer exchange. For each isolation method, ∼ 1 ml of concentrated conditioned cell medium (corresponding to the original volume of 60 ml) from cell conditioned medium batches was used. For the plasma samples both 250 μl and 3 ml sample volumes were used for each isolation method, with the exception of ExoQuick, where only 250 μl was used and Izon 35 where only 3 ml of plasma was used. The EV isolated fraction and EV‐depleted fraction were frozen and stored at ‐80°C for a maximum of 15 months.

After isolation, all the samples were suspended in the same volume, that is, 500 μl, with PBS or the appropriate buffer. Thus, each isolated sample was derived from the same original sample volume and stored in the same final volume. For the NTA and EM, 10 μl and 15 μl of the sample were used, respectively. The remainder of the sample, that is, 475 μl was used for flow cytometry analysis. For the RNA analysis, new samples were isolated from the same plasma samples and cell culture batches, and the whole sample, that is, 500 μl, was used for RNA isolation and analysis. For proteomic analysis, samples were isolated from different plasma samples and cell culture batches and the whole sample, that is, 500 μl, was used for mass spectrometry analysis. An overview is found in Figure 2 where the separate batches of plasma and cell culture supernatant are indicated with color, for example, light green and dark green for the different batches.

### 5.1 Precipitation: ExoQuick

ExoQuick ULTRA (for plasma) and ExoQuick‐TC ULTRA (for cell culture medium) (both System Biosciences) were used according to the manufacturer's protocol. In brief, 67 μl of ExoQuick was added to a maximum of 250 μl plasma sample and incubated for 30 min at 4°C. The cell culture samples were diluted in 1x PBS until a total volume of 5 ml, 1 ml of ExoQuick was added followed by an incubation overnight at 4°C. After incubation, the precipitate was spun down (3000 x g, 10 min at 4°C), resuspended in buffer and added to the purification columns. The samples were incubated for 5 min after which the EVs were eluted in a total volume of 500 μl elution buffer per isolation. The EV isolated fraction and EV‐depleted fraction were frozen and stored at ‐80°C until further use.

### 5.2 Membrane affinity: exoEasy

The exoEasy Maxi kit (QIAGEN) was used according to the manufacturer's protocol. In short, 3 ml plasma or ∼ 1 ml of concentrated cell culture supernatant was added to an equal volume of “buffer XBP” and the mixture was centrifuged (500 × g, 1 min, RT). Next, the filter was washed with “buffer XWP” and the EVs eluted in 400 μl elution “buffer XE” (500 × g for 5 min at RT, and then reapplied on the filter and centrifuged at 3000 g for 5 min at RT to maximize EV yield). All samples were topped up with elution buffer until 500 μl and stored at ‐80°C until further use.

### 5.3 Size‐exclusion chromatography: Izon 35 and 70

The 35 nm and 70 nm qEVoriginal size exclusion columns (Izon 35 and Izon 70) were used according to the manufacturer's protocol. In brief, the column was equilibrated with PBS before 500 μl of sample was loaded on top of the column. Next, the 250 μl sample was topped up with 250 μl of PBS before loading to obtain a volume of 500 μl. Subsequently, both the 250 μl and 3 ml samples were loaded onto the column in volumes of 500 μl, and 0.5 ml fractions were collected. According to the manufacturer's general use protocol, EV‐enriched fractions 7–9 were pooled. Fractions 10–12 were collected and pooled, and used as flow through control samples. The column was flushed with 20 ml filtered PBS. For the 3 ml samples, sample loading, fraction collection and column flushing was repeated until no sample was left. All fractions 7–9 were pooled together and concentrated (3000 × g for 10 min at RT) using Amicon Ultra‐4 30K centrifugal filters (Merck). All flow through fractions were pooled and concentrated (3000 × g for 10 min at RT) using Amicon Ultra‐4 100K centrifugal filters (Merck), all samples were topped up with 1× PBS until 500 μl and stored at ‐80°C until further use.

### 5.4 Iodixanol gradient: OptiPrep

Gradient fractions were prepared by diluting 60% iodixanol (Optiprep, STEMCELL Technologies) with PBS. The samples were loaded on top of an iodixanol gradient consisting of 3.5 ml 50% w/v, 3.5 ml 30% w/v and 3.5 ml 10% w/v iodixanol in open‐top thin wall polypropylene tubes (Beckman) (Karimi et al., 2018; Onódi et al., 2018). The samples were centrifuged at 130,000 × g_avg_ (SW32.1 Ti rotor, k‐factor 53, Beckman Coulter) for 16 h at 4°C with low acceleration and brake. Fractions of 0.5 ml were collected, their density was measured, and EV containing fractions (with a density of 1.12–1.19 g/ml) were pooled and diluted at least 20 times in PBS. The two closest fractions above and below the density 1.12–1.19 g/ml were separately collected and used as control samples and named OptiPrep high density (HD) and Optiprep low density (LD) fraction, respectively. Subsequently, these samples were diluted at least 60 times in 1× PBS. Diluted EVs and control samples were centrifuged in polycarbonate centrifuge bottles (Beckman Coulter) at 100,000 × g_avg_ (k‐factor 211, Ti45 rotor, Beckman Coulter) for 2 h at 4°C. The pellet was resuspended in 500 μl PBS and stored at ‐80°C until further use.

### 5.5 Protein affinity: MagCapture

MagCapture Exosome Isolation Kit PS (FUJIFILM) was used for the cell culture samples according to the manufacturer's protocol. In brief, 1 ml of sample was added to the magnetic beads and the mixture was incubated for 3 h on a rotating mixer at 4°C. Subsequently, the beads were washed three times by placing the tubes on a magnetic stand and removing the supernatant. EVs were eluted by adding 30 μl elution buffer twice. These steps were repeated until no sample was left. EV isolated fraction was topped up with elution buffer until 500 μl and supernatant were stored at ‐80°C until further use.

## 6 TRANSMISSION ELECTRON MICROSCOPY

The EVs isolated by the different methods, from both plasma and cell culture conditioned medium, were subjected to negative stain transmission electron microscopy (TEM). In brief, an aliquot of 3 μl from each sample was added to a grid with a glow discharged carbon coated supporting film for 3 min. The excess solution was soaked off by a filter paper and the grid was rinsed by adding 5 μl of distilled water for 10 s. Distilled water was soaked off by a filter paper and the grid was stained with 5 μl 1% uranyl acetate in water for 7 s. Excess stain was soaked off by a filter paper and the grid air‐dried. The samples were examined in a Hitachi HT 7700 (Hitachi, Tokyo, Japan) electron microscope at 80 kV and digital images was taken by a Veleta camera (Olympus, Münster, Germany). Particle measurement and counting was done using ImageJ 1.51w software. 200 Spherical/cup‐shaped particles were counted using at least two images with scale bar 1 μm per batch/donor of each method (600 particles in total), with exception of OptiPrep conditioned medium (262 total) and OptiPrep plasma (231 total).

## 7 NANOPARTICLE TRACKING ANALYSIS (NTA)

The particle size and concentration of obtained isolates were measured using an LM10 platform with sCMOS camera from NanoSight Ltd. The system is equipped with a 405 nm laser running NTA 2.3 analytical software package. The samples were diluted in PBS (30 kDa‐filtered) to a range of 1 × 10^8^‐1.5 × 10^9^ particles/ml and analysed with camera level 14 and detection threshold 7. For each sample, four consecutive videos were recorded in RT while injecting the sample with a syringe pump (speed 50).

## 8 BIOANALYZER

Total RNA was extracted from EVs isolated by the different methods using the miRNeasy Mini Kit (Qiagen) according to the manufacturer's protocol. The EV RNA was then analysed using capillary electrophoresis Agilent 2100 Bioanalyzer (Agilent Technologies) with the total RNA 6000 Pico Kit, according to the manufacturer's protocol.

## 9 miRNA PCR

Three batches of 60 ml of MM6 cell culture supernatant were prepared as described above, using medium containing 10% EV‐depleted FBS. Additionally, three batches of MM6 culture supernatant were prepared in which the cells were grown without FBS, referred to as serum free. From these six batches EVs were isolated by spinning the supernatant at 300 × g (10 min at RT), 3000 × g (30 min at 4°C) and for the EV collection at 120,000 × g_avg_ (2 h at 4°C), after which the EV pellets were resuspended in 300 μl PBS. Furthermore, three batches of bovine EVs were collected from the preparations of EV‐depleted medium by spinning 30% FBS (diluted in RPMI) at 100,000 × g_avg_ for 15 h at 4°C. The collected FBS EV pellets were resuspended in 2 ml PBS. From the 2 × 3 MM6 EVs and 3 FBS EVs, RNA was isolated by the miRNeasy mini kit (Qiagen), according to the manufacturer's protocol. Also from the MM6 cells RNA was isolated using the same method. Next, cDNA synthesis and RT‐qPCR was performed based on the publication by Driedonks et. al. (Driedonks et al., 2019). In short, cDNA was prepared using the miScript RT2 kit (Qiagen) with the provided HiSpec buffer. Next, using the miScript SyBR Green PCR kit (ThermoFisher) 1 ng of cDNA was used in the PCR mix along with 200 nM primer, 2.5 μl 10 × miScript universal primer and 12.5 μl of the 2 × QuantiTECT PCR Mastermix and filled up with H_2_0 to 25 μl. Cycling conditions were 95°C for 15 min followed by 40 cycles of 94°C for 15 s, 55°C for 30 s and 70°C for 30 s. After determining that mir‐122 is present in FBS but not in the MM6 cells (and thus is specific for FBS), three new MM6 cell culture batches of 400 ml were prepared and this time EVs were isolated by the six isolation methods, each method isolating from 1 ml of 30 kDa‐upconcentrated conditioned medium, representing 60 ml original volume. Next, cDNA conversion and PCR was performed again and the Cq values were analysed.

## 10 FLOW CYTOMETRY

The isolated EV fractions and flow through/supernatants were incubated with 4‐μm‐diameter aldehyde/sulfate latex beads (Invitrogen) coated with either anti‐human CD9 (clone HI9a, BD Biosciences) or anti‐human CD63 (clone H5C6, BD Biosciences) antibodies overnight at RT. Next, the beads were spun (10,000 × g, 10 min at RT) and 1 ml of 100 mM glycine was added. After a 30‐min incubation, the beads were spun down (10,000 × g, 10 min at RT) and washed with PBS containing 1% FBS. Subsequently, the beads were spun down (10,000 × g, 10 min at RT) and resuspended in PBS containing 1% FBS. A total of 1 μl (1.3 × 10^5^ beads) beads were used per staining. The beads‐EV complexes were washed in PBS (10,000 × g, 10 min at RT), incubated with PE conjugated antibodies (2 μg/ml, anti‐CD9, clone M‐L13; anti‐CD63, clone H5C6; anti‐CD81, clone 5A6; anti‐HLA‐DR, clone L243, and corresponding isotype controls, all antibodies were from BioLegend, except for anti‐CD9 that was from BD) for 30 min at 4°C, washed in PBS (3000 × g, 5 min at RT), acquired using a FACSCanto II (BDBiosciences) and analysed in Flowjo software (FlowJo LLC). Surface markers were normalized using isotype controls.

## 11 LC‐MS/MS

Forty‐five EV samples collected from either the conditioned MM6 medium or the plasma donors were analysed by label‐free mass spectrometry‐based proteomic quantification. The samples were exchanged with HEPES buffer (50 mM, pH 7.6) by concentrators in the centrifuge (5K MWCO, 4 ml, Agilent Technologies). The samples were dissolved in lysis buffer (4% SDS, 50 mM HEPES pH 7.6, 1 mM DTT) and the total protein amount was estimated by the DC protein Assay with albumin as standard, according to the manufacturer's protocol (Bio‐Rad). Protein digestion (trypsin, sequencing grade modified, Pierce) was performed using a modified protocol for SP3 protein clean up (Moggridge et al., 2018) followed by SP3 peptide clean up. Each sample was separated using a Thermo Scientific Dionex nano LC‐system in a 3 h 5–40% ACN gradient coupled to Thermo Scientific High Field QExactive‐HF. Spectra were searched using MSGF+ and Percolator within a proteomics analysis pipeline developed in Nextflow (https://github.com/lehtiolab/ddamsproteomics, vs1.5) against the human protein database Ensembl 99. Protein identification results were filtered at a 1% false discovery rate limit. Protein quantification was performed by MS1 feature detection, using the average of the top‐3 highest intensity peptides for MS1 protein quantification. For peptide quantification, the highest MS1 intensity peptide‐spectrum‐match (PSM) was used.

## 12 PROTEOMIC ANALYSIS

The quantified proteins were analysed using R (Rstudio, version 1.1.383). To increase confidence in protein quantitative accuracy, only proteins with Peptide‐Spectrum Match scoring above three that were present in at least two of the five samples (three plasma donors, two MM6 cell medium batches) were selected for further analysis. Subsequently, the intensities of these proteins were log2 transformed and used for downstream analyses.

The Principal Component Analysis (PCA) and Hierarchical Agglomerative Clustering (HC) were performed using inverse correlation as distance matrix and Ward.D2 as linkage method. Proteins belonging to each cluster in the HCA were subjected to Geno Ontology (GO) enrichment analysis using the package “enrichR” (Kuleshov et al., 2016) with the “GO_Cellular_Component_2018″ gene set (The Gene Ontology, 2019).

To classify plasma and EV proteins, three protein sets were created and the proteins in these three lists were matched with dataset from this study. For the Vesiclepedia top 100 list (Table S1), version 4.1 was downloaded from the Vesiclepedia database on August 15, 2018, http://microvesicles.org, and proteins were limited to Homo Sapiens. For vesicle‐related GO list (Table S2), the GO Cellular Component dataset from MSigDB (Subramanian et al., 2005) was downloaded on September 28, 2020. Proteins present in the Cellular Compartment GO terms associated with the Endosomal Sorting Complexes Required for Transport (ESCRT), MVB, and vesicle pathways (*n* = 35) were selected, summarising the current knowledge on proteins related to membrane budding and vesicle transport (Table S2). The plasma protein list, composed to represent “typical plasma proteins”, was based on a previous publication (Anderson & Anderson, 2002), and this list was further supplemented with all the IGLV and IGKV light chains identified in the dataset from this study (Table S3).

## 13 STATISTICS

Multiple groups were compared by one‐way ANOVA followed by a multiple comparison analysis with Tukey's multiple comparison test. Grouped values of multiple groups were analysed by two‐way ANOVA followed by a multi comparison analysis with Tukey's multiple comparison test. *P*‐values < 0.05 were considered as statistically significant. Analyses were performed with GraphPad software version 8.3.

For the PCA's the allocation of each observation to the nearest cluster was performed by K‐means clustering, with the number of clusters set to 4. Ellipses were drawn around the centroids of the clusters, representing 95% confidence intervals.

## 14 RESULTS

To compare the total EV yield of the different isolation methods and their efficiency to enrich for EVs versus non‐EV entities, EVs were isolated from three human plasma samples and three batches of conditioned medium derived from MM6 cells. The isolated EV fractions were subjected to multiple analyses, that is, transmission electron microscopy (TEM), Nanoparticle Tracking Analysis (NTA), Bioanalyzer, Flow cytometry, and LC‐MS/MS (Figure 2).

### 14.1 Distinct heterogeneous populations are isolated by the different EV isolation methods

**FIGURE 3:**
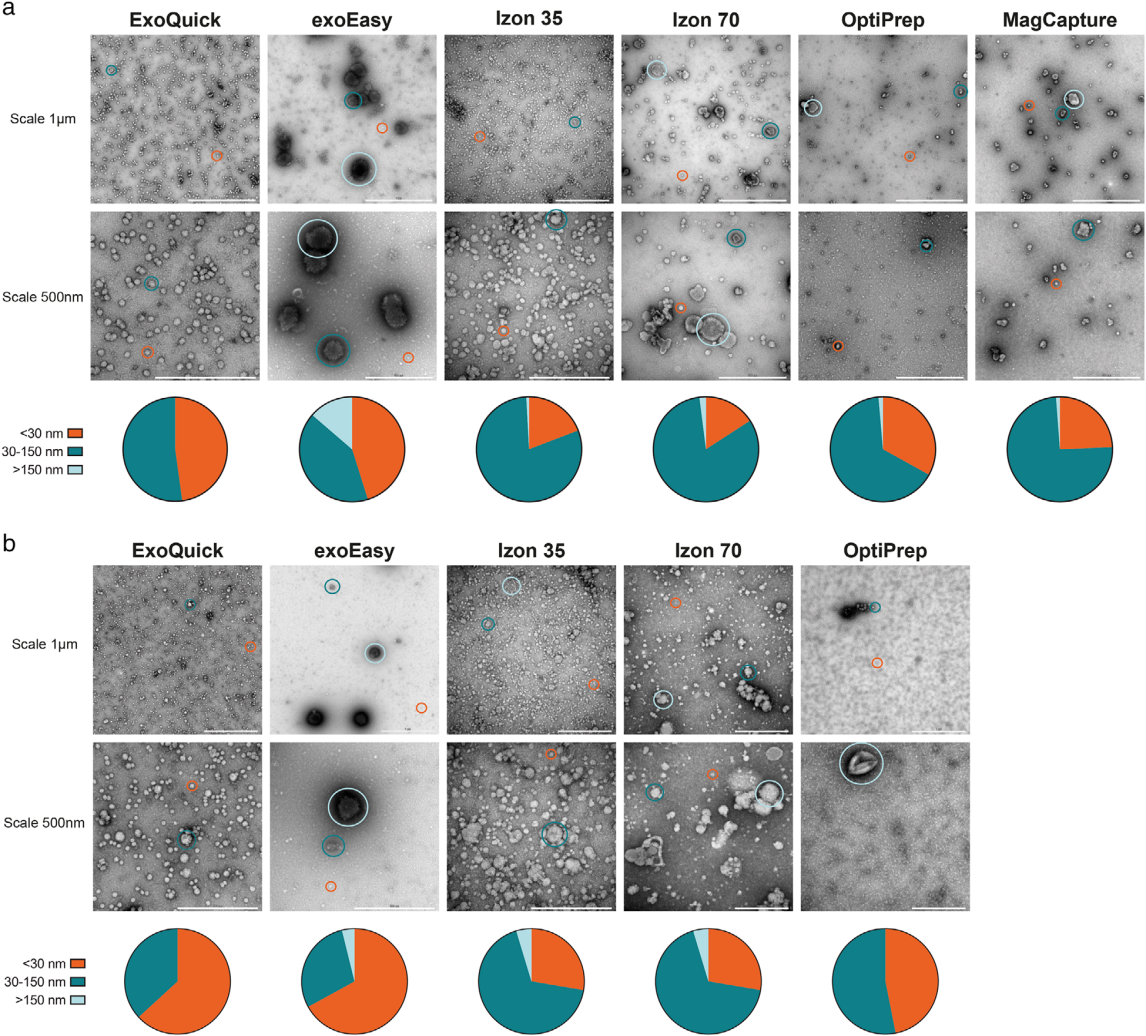
Morphological characterization of conditioned medium‐ and plasma isolated EVs. Morphological analysis by negative stain transmission electron microscopy (TEM) of isolated particles using ExoQuick, exoEasy, Izon 35, Izon 70, OptiPrep and MagCapture from (a) conditioned medium from MM6 cells and (b) human plasma. Scale bars are 1 μm on the top rows and 500 nm on the bottom rows. Representative spherical or cup‐shaped particles of different sizes are circled. Orange circles indicate non‐EV particles < 30 nm, green circles indicate 30–150 nm sized particles, blue circles indicate > 150 nm sized particles. All images are representative TEM images out of at least 10 images analysed from each EV isolate sample (from each batch/donor and isolation method). Pie charts represent the size distribution of up to 200 spherical or cup‐shaped particles counted using at least 2 EM images per batch/donor.

First, we sought to compare isolation methods based on the morphology and size distribution of the isolated particles. Therefore, negative stain TEM analysis of the EV containing fractions was performed. Note, since vesicles with a lipid bilayer, that is, EVs, are physically unable to be smaller than 30 nm (Huang et al., 2017), particles seen in the TEM smaller than 30 nm were, therefore, considered as co‐isolated entities. These entities can be, for example, smaller lipoproteins (HDL/LDL/IDL), exomeres, or protein and protein complexes seen as the amount of granularity of the background. Consequently, we have not only indicated EVs in Figure 3.

The EM results showed a great heterogeneity between the different isolation methods, regarding both EV subpopulation and lipoproteins (Figure 3a and b). Spherical or cup‐shaped particles < 30 nm were considered lipoprotein, 30—150 nm small EVs or lipoprotein and > 150 nm large EVs or lipoprotein. For both conditioned medium and plasma, *Izon 35* and *Izon 70* seem to isolate the most heterogeneous population. Furthermore, *exoEasy* isolates a larger population of phenotypically larger cup‐shaped EVs compared to the other methods, visually indicating a clearly different subpopulation. In contrast, *ExoQuick* appears to enrich for smaller particles, presumably lipoproteins. For *OptiPrep* only few particles could be identified in the plasma samples. Non‐EV enriched fractions of the two *Izon* columns showed many particles, and the later fractions high granularity, indicating high protein. For the conditioned medium samples of *Izon 70*, there were some cup‐shaped particles in the early flow through fractions, and for the non‐EV fractions of *OptiPrep* there were some present in the lower‐density fractions (Figure S1A). The high‐density fractions of *OptiPrep* of the plasma samples mainly contained protein complexes, with some spherical particles (around 60 nm) found in the low‐density fractions (Figure S1B).

It is generally difficult to distinguish EVs from lipoproteins in negative stain TEM. To evaluate the contribution of added serum in the conditioned medium and to possibly differentiate between lipoproteins and small EVs, EM on particles/EVs isolated by *Izon 70* from cells grown in serum free medium for 48 h, and non‐conditioned culture medium containing 10% EV‐depleted serum, was performed (Figure S1C). From these images, we concluded that it is virtually impossible to morphologically distinguish lipoproteins from small EVs by TEM in this setting.

### 14.2 The different EV isolation methods resulted in different size distributions, particle number and protein amount

**FIGURE 4:**
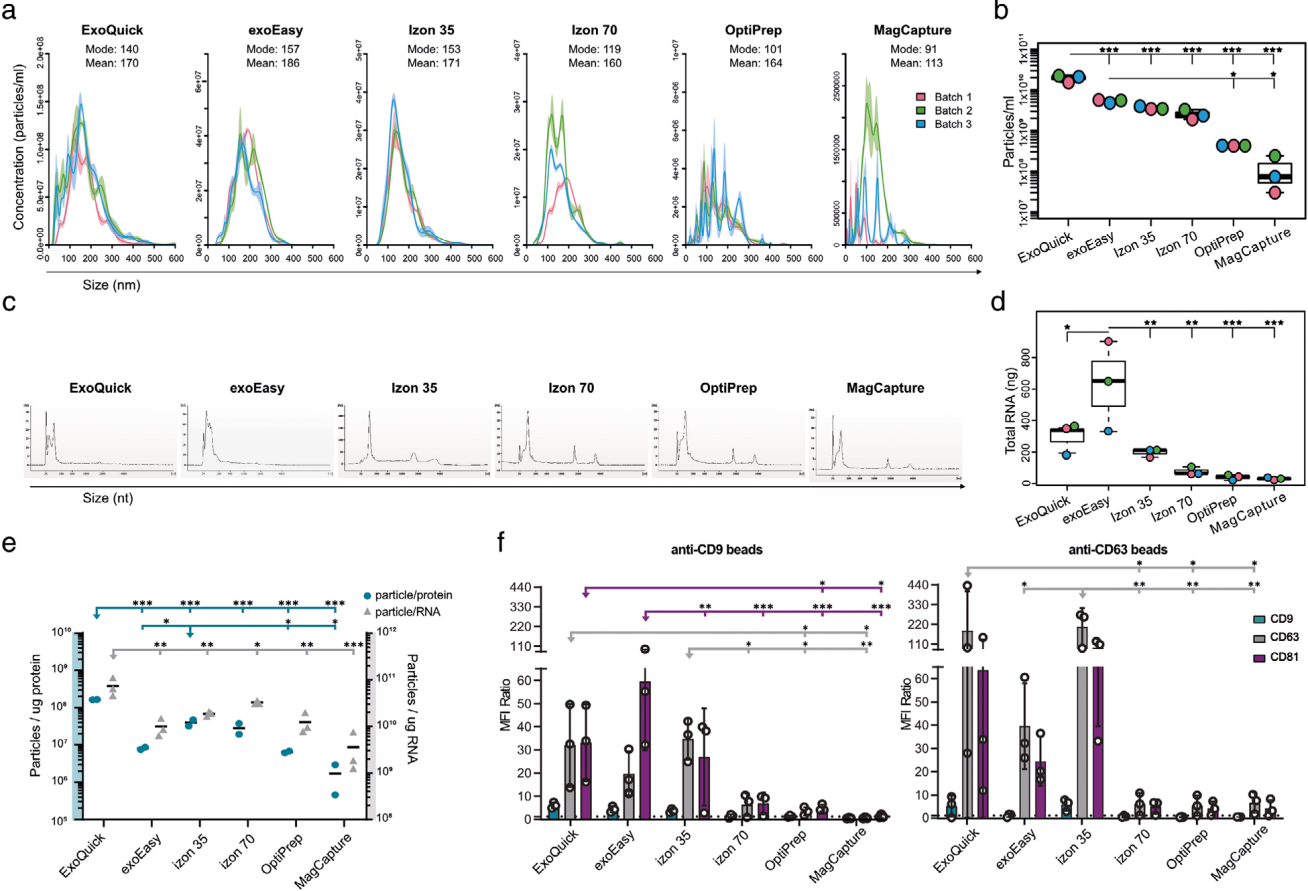
Characterization of conditioned medium derived EVs. Phenotypic analysis of EV isolates using five different EV isolation methods (ExoQuick, exoEasy, Izon 35, Izon 70, OptiPrep and MagCapture) from three 60 ml‐batches of conditioned media from MM6 cells. (a) Size distribution (nm) of isolated particles and (b) particle count (per ml) of conditioned medium using Nanoparticle Tracking Analysis. (c) Bioanalyzer analysis of total RNA. Representative electropherograms (*n* = 3) show the size distribution in nucleotides (nt) and fluorescence intensity (FU) of total RNA. The peak at 25 nt is an internal standard. (d) Total amount of isolated RNA in ng. Data analysed by one‐way ANOVA with Tukey's HSD for multiple comparisons. Dots represent a single batch, data presented as mean ± SD. *P < 0.05, **P < 0.01 and ***P < 0.001. (e) The total amount of particles was divided by the total amount of protein (right y‐axis) or total amount of RNA (left y‐axis), creating ratio plots. Data analysed by one‐way ANOVA with Tukey's HSD for multiple comparisons. Dots represent a single batch, data presented as mean. *P < 0.05, **P < 0.01 and ***P < 0.001. (f) EVs were bound to anti‐CD9 or anti‐CD63 coated latex beads and analysed using flow cytometry to show the presence of the surface markers CD9 (blue), CD63 (grey) and CD81 (purple) on EVs. Data is shown as MFI ratio between specific antibody and the corresponding isotype control. A signal above 1 (dotted line) was considered as a positive signal. Data analysed by two‐way ANOVA with Tukey's HSD for multiple comparisons. Dots represent a single donor, data presented as mean ± SD. *P < 0.05, **P < 0.01 and ***P < 0.001

**FIGURE 5:**
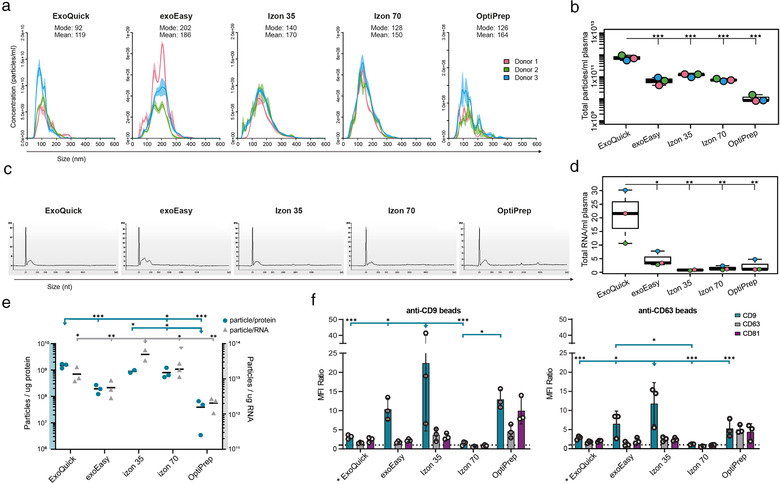
Characterisation of plasma derived EVs. Phenotypic analysis of EV isolates using four different EV isolation methods (ExoQuick, exoEasy, Izon 35, Izon 70 and OptiPrep) from plasma of three heathy donors. (a) Size distribution (nm) of isolated particles and (b) particle count (per ml) using Nanoparticle Tracking Analysis. (c) Bioanalyzer analysis of total RNA. Representative electropherograms (*n*  =  3) show the size distribution in nucleotides (nt) and fluorescence intensity (FU) of total RNA. The peak at 25 nt is an internal standard. (d) Total amount of isolated RNA in ng per ml of plasma. Data analysed by one‐way ANOVA with Tukey's HSD for multiple comparisons. Dots represent a single donor, data presented as mean ± SD. *P < 0.05, **P < 0.01 and ***P < 0.001. (e) The total amount of particles was divided by the total amount of protein (right y‐axis) or total amount of RNA (left y‐axis), creating ratio plots. Data analysed by one‐way ANOVA with Tukey's HSD for multiple comparisons. Dots represent a single batch, data presented as mean. *P < 0.05, **P < 0.01 and ***P < 0.001. (f) EVs were bound to anti‐CD9 or anti‐CD63 coated latex beads and analysed using flow cytometry to show the presence of the surface markers CD9 (blue), CD63 (grey) and CD81 (purple) on EVs. Data is shown as MFI ratio between specific antibody and the corresponding isotype control. A signal above 1 (dotted line) was considered as a positive signal. Data analysed by two‐way ANOVA with Tukey's HSD for multiple comparisons. Dots represent a single donor, data presented as mean ± SD. *P < 0.05, **P < 0.01 and ***P < 0.001

To further evaluate the size distribution and in addition the number of the isolated particles from the different EV isolation methods, NTA was performed. Overall, the NTA results, in concordance with the EM results, show a broad size distribution of particles for all methods. However, for the conditioned medium samples (Figures 4a and 4b), the NTA analysis of the *ExoQuick* samples showed quite a different representation compared to the EM and plasma NTA analysis. Where in EM no larger particles could be seen, here a broad size range could be observed from 30 to 350 nm (Figures 4a and 5a). In addition, for both sample types, this method showed a significantly higher particle count /ml compared to the other methods (Figures 4b and 5b). The *exoEasy* samples were in line with the EM data, showing larger particles than the rest of the methods for both medium and plasma (Figures 4a and 5a). Both the *Izon 35* and the *Izon 70* samples showed a broad size range of 50—300 nm with *Izon 35* isolating slightly more particles than *Izon 70* (Figures 4b and 5b). While these methods look very similar in size distribution, *Izon 35* appears to isolate more of the larger vesicles than *Izon 70*, for both sample types (Figures 4a and 5a). For both the *OptiPrep* and *MagCapture*, no appropriate estimates of mode size could be made as both samples had a very low particle concentration, backed by the EM results, but most particles were below 300 nm for both types of samples (Figures 4b and 5b).

In addition to particle number, the total protein amount (yield in μg) in the EV isolates was estimated for both the plasma and conditioned medium samples (Tables 1 and 2). For the conditioned medium the *exoEasy* samples resulted in the highest amount of isolated proteins (Table 1). Also for the plasma samples *exoEasy* was the method that resulted in the highest protein amount together with *OptiPrep* (Table 2). Remarkably, EV isolation of Donor 3 by *OptiPrep* resulted in a 10‐fold higher protein yield, which could be either due to donor variability only detected by this method, experimental error, or low reproducibility of the method.

**TABLE 1:** Total protein amount in each batch and isolation method for the conditioned cell medium‐derived EVs

	Total proteins (μg)					
	ExoQuick	exoEasy	Izon 35	Izon 70	OptiPrep	MagCapture
MM6 batch 1	95	662	73	99	70	66
MM6 batch 2	136	745	108	88	63	81

**TABLE 2:** Total protein amount in each donor and isolation method for the plasma‐derived EVs

	Total protein (μg)							
	250 μl				3 ml			
	ExoQuick	exoEasy	Izon 70	OptiPrep	exoEasy	Izon 35	Izon 70	OptiPrep
Plasma D1	29	121	11	31	344	121	99	166
Plasma D2	69	108	12	11	347	135	68	231
Plasma D3	34	112	26	25	334		132	2457

Interestingly, the total protein amount did not naturally correlate with particle numbers, since for both conditioned medium and plasma *ExoQuick* was the method that isolated most particles, but not the highest protein amount, resulting in a significantly higher particle‐to‐protein ratio compared to the other methods, for both conditioned medium and plasma (Figures 4e and 5e). Furthermore, for the plasma samples, *Izon 35* had significantly higher protein‐to‐particle ratios compared to all other methods, but not a significantly higher particle number (Figure 5e). In contrast, for the conditioned medium, *exoEasy* isolated significantly more particles than *OptiPrep* and *MagCapture*, around 15‐fold and 40‐fold more, respectively. But *exoEasy* also isolated a higher amount of protein, around 630 μg more, resulting in similar particle‐to‐protein ratios between these three groups (Figure 4e).

### 14.3 All EV isolation methods isolate RNA with classical EV‐like RNA pattern

To evaluate the RNA yield and RNA size distribution of the isolated EV samples from the different EV isolation methods, total RNA was analysed using an Agilent 2100 Bioanalyzer. For both the conditioned medium and plasma samples, all methods resulted in a classical EV‐like RNA pattern with an enrichment of smaller RNA (Figures 4c and 5c). For both EV sources, the RNA yield varied between the different EV isolation methods. The total RNA amount for conditioned MM6 cell medium‐derived EVs ranged from 628 ng in *exoEasy* to 32 ng in *MagCapture*, with *exoEasy* isolating significantly more RNA than the other methods (Figure 4d). For the plasma‐derived EVs, when these numbers were normalised to plasma volume, the yield ranged from 20.8 ng/ml plasma for the *ExoQuick* and 0.8 ng/ml plasma for *Izon 35*, with *ExoQuick* isolating significantly more RNA than the other methods (Figure 5d).

To investigate the presence of FBS‐derived RNA in the EV isolates, the samples were tested for the presence of miR‐122‐5p, which has previously been described not to be depleted by overnight spinning of FBS (Lehrich et al., 2019). Indeed, while miR‐122 was significantly lower in the MM6‐derived EVs isolated by ultracentrifugation compared to the 30% FBS pellet, miR‐122 was still present in higher levels compared to the MM6‐derived EVs cultured in FBS‐free medium, although this result was not significant (Figure S2A). When miRNA‐122 expression was analysed in the EV fractions from the different isolation methods, all methods isolated less miRNA‐122 compared to EVs isolated by ultracentrifugation, although not significantly (Figure S2B). These results suggest that none of the isolation methods enrich for bovine‐derived miR‐122‐5p.

Interestingly, when correlating RNA to particle numbers (Figures 4e and 5e), some correlation could be seen but not for all methods. For conditioned medium *ExoQuick* had the highest amount of particles, but not the highest RNA yield, resulting in a significantly higher particle‐to‐RNA ratio than the other methods (Figure 4e). Furthermore, *exoEasy* had the highest RNA yield, but not a significantly higher particle number, resulting in a low particle‐to‐RNA ratio. This was supported by EM‐pictures which showed low particle numbers. For *OptiPrep* and *MagCapture* the results correlated, as they had both the lowest particle number and RNA yield, resulting in a low particle‐to‐RNA ratio (Figure 4e). For the plasma samples, the particle number of *ExoQuick* was shown to correlate with the total RNA yield (Figures 5b and d), which it did not do for the conditioned medium. Furthermore, *Izon 35* had the significantly highest particle‐to‐RNA ratio for the plasma samples, supported by a large number of particles seen in EM‐pictures.

### 14.4 All EV isolation methods showed an enrichment for the common EV enriched tetraspanins by flow cytometry

To evaluate the presence of the EV enriched tetraspanins CD9, CD63, and CD81, the EV isolates were analysed by flow cytometry using anti‐CD9 or anti‐CD63 coated latex beads. The conditioned MM6 cell medium samples isolated by *ExoQuick*, *exoEasy* and *Izon 35* showed significantly higher signals for either CD63 or CD81 (both for *ExoQuick)* on the anti‐CD9 beads, while for the anti‐CD63 beads *ExoQuick* and *Izon 35* expressed significantly higher levels of CD63 than the other EV isolation methods (Figure 4f). For the plasma samples *exoEasy* and *Izon 35*, with the addition of *OptiPrep*, showed the significantly higher signals for CD9 in comparison to the other methods (Figure 5f). Note that for *ExoQuick* only 250 μl of plasma was used, and still a slight positive signal for all three tetraspanins was seen (Figure 5f). For the conditioned medium samples, the isolation methods *Izon 70*, *OptiPrep* and *MagCapture* showed only low signals for the tetraspanins (Figure 4f), and for the plasma samples *Izon 70* did not express detectable levels of any tetraspanins (Figure 5f). Moreover, for the conditioned medium samples isolated by *MagCapture*, all three tetraspanins were negative on the anti‐CD9 beads while some signal could be detected on the anti‐CD63 beads (Figure 4f).

The ratios between the three tetraspanins varied slightly between the different methods, indicating that the relative abundance of subpopulations of EVs were different. Interestingly, for conditioned MM6 cell medium the vesicles captured on the anti‐CD9 beads, *ExoQuick*, *exoEasy*, and *Izon 35* detected roughly the same levels for the three tetraspanins. However, for the vesicles captured on the anti‐CD63 beads, *exoEasy* showed much lower presence of the three tetraspanins compared to *ExoQuick* and *Izon 35*, indicating that *ExoQuick* and *Izon 35* enrich for other types of vesicles.

In addition, since *Izon* is based on size and *OptiPrep* is based on density, the separately collected fractions enrich for different particles. The “EV fractions” will be enriched for EVs, while the supposedly non‐EV fractions are enriched for particles smaller, or with different densities, respectively. To determine if the low presence of tetraspanins in the *Izon 70* and *OptiPrep* samples for the conditioned medium was due to that more EVs were present in the EV‐depleted fractions, these fractions were analysed (Figures S3A and B). Indeed, for the conditioned MM6 cell medium, the flow‐through fractions of the *Izon 70* samples showed a high signal for both CD63 and CD81 on the anti‐CD63 beads, although not significant. For the plasma samples, of which the EV fractions were negative for all markers on both beads, the flow‐through fractions showed a significant signal for CD9 and CD63. This indicates that more EVs were present in the flow‐through than in the EV fraction, suggesting that all fractions should be evaluated when using this method. In contrast, the *OptiPrep* EV‐depleted fraction of conditioned medium, showed no signal in either the low or high‐density fraction for the anti‐CD9 beads. However, there was a low signal for CD63 and CD81 on the anti‐CD63 beads (Figure S3A). For the plasma, however, the low‐density fraction of the *OptiPrep* showed high positive signals for all three tetraspanins on the anti‐CD9 beads although not significant, indicating that some EVs might float at a lower density than suggested (Figure S3B). In addition, also the flow‐through fractions of *Izon 35* showed a positive signal of tetraspanins for both EV sources on the CD63 beads (Figures S3A and B).

### 14.5 Mass spectrometry‐based proteomics confirms different EV populations isolated by the individual methods

Since the different methods isolated EVs with distinct morphology, protein, and RNA yields, we investigated if they could also impact the proteomic profile of the isolated fraction. To this end, we performed LC MS/MS on all the EV–enriched fractions and the flow‐through fractions of the *Izon 35* and *Izon 70*. Since the flow‐through fractions should have relatively fewer EVs and more non‐EV proteins such as lipoproteins, these fractions were included in the following analysis to determine whether the different methods actually enrich for EVs. Next, proteomic analysis was performed. First, an unsupervised cluster analysis was performed on the samples to visualise potential batch effects, since the *Izon 35* samples were isolated from different cell batches. As seen in the Principal Component Analysis (PCA), the different batches clustered together, indicating no batch effect (Figure S4A). Summaries of the number of identified proteins for each sample is provided in Tables 3 and 4.

**TABLE 3:** Number of identified proteins in each batch and isolation method for the conditioned cell medium‐derived EVs

		Conditioned cell medium 60 ml					
		ExoQuick	exoEasy	Izon 35	Izon 70	OptiPrep	MagCapture
Identified proteins per batch	MM6 batch 1	102	530		537	330	487
	MM6 batch 2	233	497		703	234	635
	MM6 batch 3			881			
	MM6 batch 4			934			
	Total identified proteins	248	590	1014	806	387	714
	Unique proteins	2	49	96	0	1	15

**TABLE 4:** Number of identified proteins in each donor and isolation method for the plasma‐derived EVs

		Plasma 250μl				Plasma 3 ml			
		ExoQuick	exoEasy	Izon 70	OptiPrep	exoEasy	Izon 35	Izon 70	OptiPrep
Identified proteins per donor	Plasma D1	209	207	26	139	264	199	258	171
	Plasma D2	183	236	82	144	279	194	260	140
	Plasma D3	160	215	74	132	239		280	149
	Total identified proteins	243	273	109	174	311	225	335	194
	Unique proteins	1	5	0	0	10	1	4	2

**FIGURE 6:**
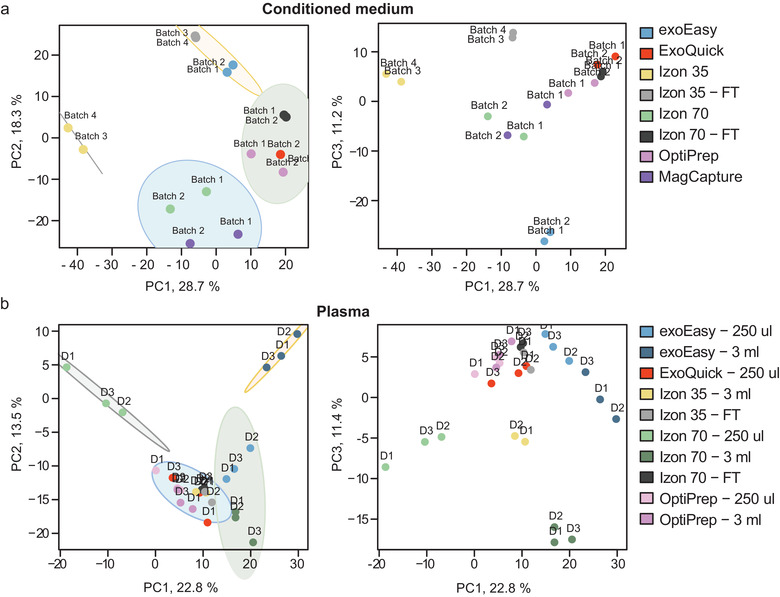
Different isolation methods result in diverse EV samples. Principal Component Analysis (PCA) was performed on the identified proteins by mass spectrometry. The variance contained by the stated PC is shown as percentage, ellipses represent 95% CI. (a) PCA on proteins identified in the conditioned medium samples. The first plot shows PC1 versus PC2 and the second plot shows PC1 versusversus PC3. (b) PCA on proteins identified in the plasma samples. The first plot shows PC1 versus PC2 and the second plot shows PC1 versus PC3.

Sample comparison using PCA showed that the plasma samples cluster together while the conditioned MM6 cell medium derived samples cluster more separately from each other (Figure S4B). A likely explanation is because the plasma samples have more abundant soluble plasma proteins than the conditioned medium samples, 41 versus 13 plasma proteins on average per sample, which result in a decreased complexity of the samples and, therefore, a tighter cluster in the PCA. Indeed, while for the conditioned medium the flow‐through fractions of the *Izon 35* and *70* columns separated from the EV fractions of *Izon 35*, *Izon 70* and *MagCapture* (Figure 6a), most plasma EV fractions clustered together with these flow‐through fractions (Figure 6b). For the plasma samples, a clear separation of the 250 μl *Izon 70* samples was seen in the PCA which can be explained by the low protein counts of these samples (Figure 6b). Interestingly, the *exoEasy‐ 3 ml* samples clustered separately from the other plasma samples. This result was also visible in the PCA on the medium samples where the *exoEasy* samples separated from the other EV samples (Figure 6a). This separation indicates that *exoEasy* enriches for a distinct set of proteins/EVs, which is supported by the previous EM data where larger vesicles were identified. Additionally, for the conditioned medium, the *Izon 35* samples showed to be the most distinctive compared to the other samples seen by the separation by PC1, which can be explained by the fact that this method contained the most unique proteins (Table 3).

### 14.6 Clear separation of EVs from plasma proteins is challenging for plasma samples, while for the conditioned medium the EV fractions show a distinct EV enrichment depending on the isolation method

To determine if the isolation methods enrich for EV proteins compared to soluble proteins and other particles, and whether this enrichment is sample‐volume depended, we compared the identified proteins from the proteomics experiment with public domain data.

Three different datasets were used to represent different potential protein components in the samples. We used two separate EV protein datasets called: The Vesiclepedia top 100 list and the vesicle‐related GO list. The third dataset consisted of a list with common plasma proteins (a description of how the lists were created is in the Material and Methods).

**FIGURE 7:**
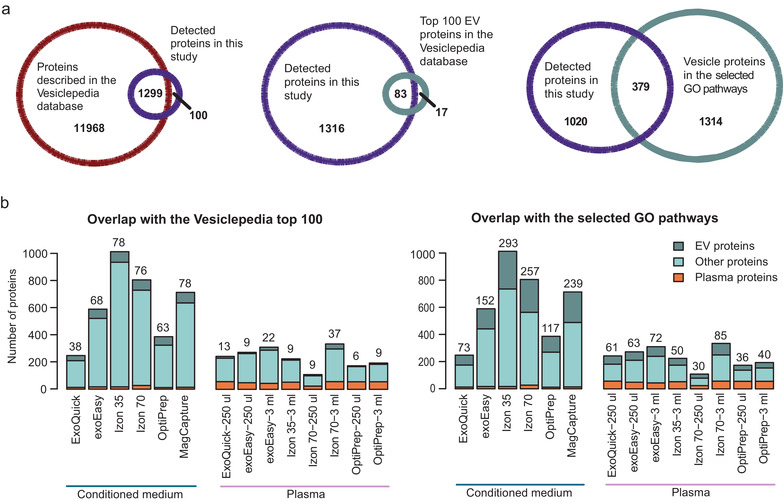
EV yield and purity by different isolation methods. The number of EV‐related proteins and plasma proteins were calculated for each isolation method by comparing the protein overlap with two distinct EV protein reference lists and a plasma protein list. (a) First, the total number of identified proteins in this study were compared to all proteins in the Vesiclepedia database. Then, the identified proteins in this study were compared to two EV protein lists. The first list contains the top 100 reported proteins in the Vesiclepedia data base (supplementary list 2). The second list is built from the proteins present in the Cellular Compartment GO terms related to ESCRT, Multivesicular body and Endosome (supplementary list 3). (b) The identified proteins isolated by each method were compared to the plasma protein list (supplementary list 1), and matched proteins were categorised as plasma proteins. Next, the non‐plasma proteins were compared to both the “Vesiclepedia top 100″ list and the “selected GO pathway” list and overlapping proteins were labelled as EV proteins. Proteins belonging neither to plasma or EV proteins were categorised as “other proteins”. The number of identified EV proteins for each method were also stated above the corresponding bar

First, we compared all the detected proteins in our study with the complete Vesiclepedia database. The majority of proteins that were detected in our study were also shown to be present in this database. Interestingly, 100 proteins that were found in our study have not yet been described in the Vesiclepedia database (Figure 7a). Of these 100 proteins, 33 were IgG light chain variables and interestingly also MVB12A and MVB12B, which are components of the ESCRT‐I, an important protein complex for membrane remodelling and vesicle formation. After a manual check in the Vesiclepedia database, these proteins were actually present in the database but listed by their old gene names FAM125a and FAM125b. This discrepancy indicates that the Vesiclepedia database should be used carefully as it is not updated to the latest HGNC‐approved nomenclature.

Next, we examined the overlap between the identified proteins in our study with both EV protein lists (Figure 7a). Of the top 100 most reported proteins in Vesiclepedia, 83 were found in this study. The 17 proteins that were not detected in this study included among others nine proteins from the histone H4 family and FLOT1. The EV protein list build with the vesicle‐related GO terms contains many more proteins, that is, 1693, and therefore, a larger overlap of 379 proteins was found between this list and the proteins identified in this study.

Following the illustrative overlap analyses between this study and the two EV protein lists, we calculated the number of identified EV‐ and plasma proteins for each isolation method, for both the conditioned medium and the plasma samples using both of the EV protein lists (Figure 7b). In this and further analyses, we did not include the flow‐through fractions, as we are comparing EV isolation methods and these fractions are not intended to isolate EVs. Importantly, both the Vesiclepedia top 100 list and the vesicle‐related GO list contain plasma proteins that were also present in the used plasma protein list (3 and 15 proteins, respectively). Therefore, all proteins detected in each isolation method were first compared to the plasma list, and any matching proteins were categorised as a plasma protein. Then, the non‐plasma classified proteins in each isolation method were compared to the two EV protein lists and overlapping proteins were labelled as EV proteins. Lastly, proteins that were absent in both the plasma list and the EV protein lists were then named as other proteins. If a protein was expressed in multiple donors or batches of one isolation method, it would not count double or triple. Hence, the protein numbers in Figure 7b represent the counts of different proteins identified and not counts of different proteins and how often they were identified.

When looking at the conditioned medium samples, the Vesiclepedia top 100 list identified both *Izon 35* and *MagCapture* (both 78 EV proteins) as the methods that isolated most EV proteins, followed by *Izon 70* (76 EV proteins). The vesicle‐related GO list showed that *Izon 35* isolated most EV proteins, followed by *Izon 70* and thirdly *MagCapture*. Although *Izon 70* isolated many EV proteins, it was also the method that isolated the most plasma proteins: 27 proteins, followed by 17 proteins in *exoEasy* and *Izon 35*. For the plasma samples *Izon 70 – 3 ml* was also the method that isolated the most EV proteins (37 and 85 proteins) but it did not isolate the most EV proteins when smaller volumes were used. *exoEasy – 3 ml* isolated the second most EV proteins, independent of which EV list that was used. Moreover, *Izon 70 – 3 ml* also isolated slightly more plasma proteins (57 proteins) and total proteins than *exoEasy ‐ 3 ml* (44 plasma proteins). In contrast, *Izon 35* isolated fewer EV proteins (nine and 35 proteins) and fewer total proteins, but equal amounts of plasma proteins (53 proteins), indicating an enrichment of plasma proteins by this method. In addition, out of the 3 ml plasma samples, *exoEasy* was shown to isolate the least plasma proteins. Interestingly, while *ExoQuick* isolated a high amount of EV proteins for the 250 μl plasma samples, independently on the EV protein list used, this method isolated the lowest number of EV proteins and lowest amount of total protein for the conditioned medium samples. Furthermore, this method also isolated the most particles (Figures 4b and 5b), suggesting that many non‐EV particles are isolated by this method. The methods that isolated the least EV proteins in plasma were *Izon 70 ‐ 250 μl* (nine and 30 proteins) and *OptiPrep – 250 μl* (nine and 36 proteins), depending on which EV list that was used for the comparison. These results might be explained by the fact that the total amount of protein (yield in μg) was low in these samples. Furthermore, *OptiPrep* isolated similar amounts of plasma proteins as the other methods (56 proteins for both volumes), while in comparison it isolated the least EV proteins, indicating that *OptiPrep* co‐isolates many plasma proteins.

### 14.7 Hierarchical clustering and GO annotation analysis show enrichment of vesicle related proteins and pathways for all isolation methods

**FIGURE 8:**
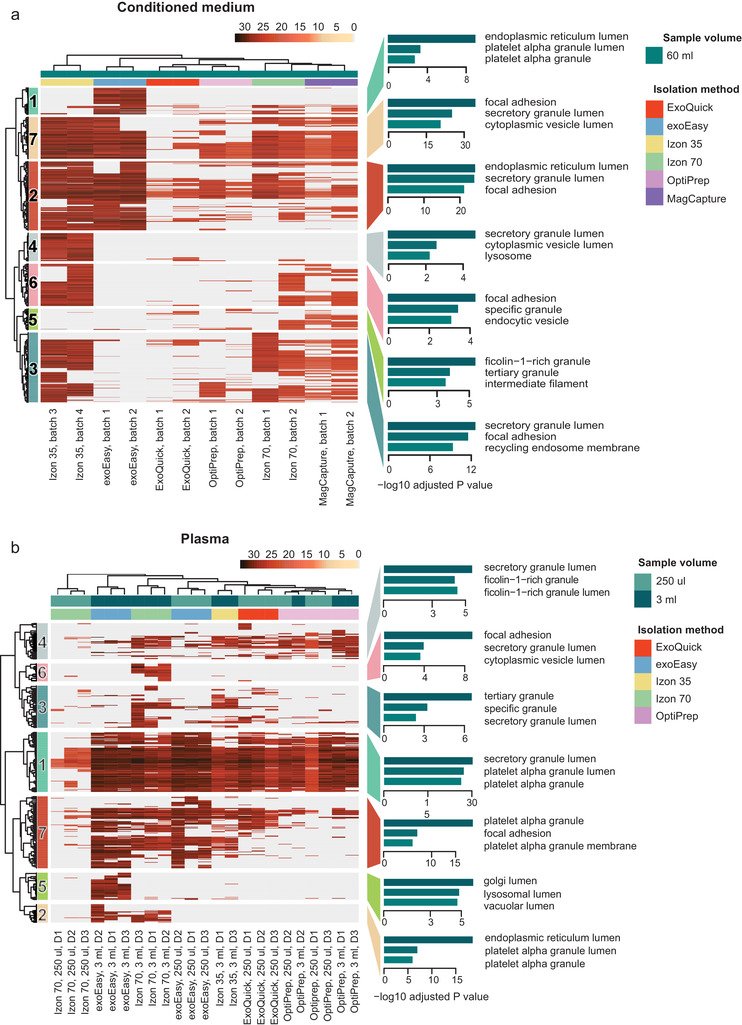
HCA and GO enrichment of the subsequent identified clusters show that all isolation methods enrich for vesicles. Hierarchical Clustering Analysis (HCA) and subsequent GO enrichment for Cellular Components on the HCA identified clusters was performed for isolated EV samples from (a) conditioned medium from MM6 cells and (b) human plasma. The scale of the HCA shows log2 transformed intensities of the proteins. Absent proteins are displayed in grey.

After determining the amount of EV proteins isolated by each method and the purity of these EV fractions, we wanted to determine what type of proteins each method isolates using Gene Ontology (GO) analyses. To explore this, we performed Hierarchal Clustering Analysis (HCA) and on the identified protein clusters we performed GO enrichment analysis to identify which GO terms are represented for each cluster. In general, the GO annotation showed that all isolation methods enrich for vesicle related proteins as shown by terms such as “secretory granule lumen”, “cytoplasmic vesicle lumen”, and “tertiary granule” indicating that the methods all enrich for EVs, independent of sample origin (Figures 8a and b).

The heatmap visualising the clustering of the conditioned medium‐derived EV samples shows a similar clustering pattern as the PCA (Figure 6a), as for both analyses the *Izon 35* method clusters furthest apart from the other samples, followed by the *exoEasy* method (Figure 8a). Moreover, as shown above (Figure 7b), the *ExoQuick* method yield the lowest number of proteins as seen by the absence of proteins in most clusters, visualised by a grey colour (Figure 8a).

Similar to the medium samples, the hierarchical clustering analyses of the plasma data (Figure 8b) shows a similar pattern as the PCA (Figure 6b), in which the *Izon 70–250 μl* samples also cluster most separately. This separation is likely due to the low protein amount in these samples as identified before (Table 2) and here visualised by the grey colour. The sample load appears to be important, as for most methods the samples within the isolation methods cluster separately based on their volume, except for the *OptiPrep* method, where the 250 μl and 3 ml samples cluster together. Given that higher starting volumes of the samples can increase the amount of isolated EVs as compared to co‐isolated plasma proteins (Figure 7b), this separation of the high volumes might be a result of that they contain relatively more EV proteins. Consequently, the mass spectrometer might be able to detect these scarce EV proteins which would otherwise be masked by the abundant plasma proteins.

For the conditioned medium samples several EV proteins such as CD81, Alix, RAB proteins, integrins, and ICAM were found in cluster 7, while other EV proteins such as CD9, CD63 and TSG101, were found in cluster 3 (Figure 8a). In addition, ESCRT II protein VPS25, ESCRT III proteins CHMP2A, CHMP3, CHMP4B and CHMP6, ESCRT III associated protein VPS4B and RAB proteins were also described in cluster 3, indicating an endosomal origin. Interestingly, for the medium samples, cluster 7 was enriched by all isolation methods, except for *ExoQuick* and to a lesser extent *OptiPrep*. This was also the case for cluster 3 except for *ExoQuick, OptiPrep, and exoEasy*, indicating that *exoEasy* enrich for vesicles (cluster 7) but not specifically for ESCRT enriched vesicles (cluster 3). Overall, it appears that *ExoQuick* and *OptiPrep*, and to a lesser extent *exoEasy*, do not enrich for the classical EV proteins from conditioned medium.

For the plasma samples the classical EV proteins such as tetraspanins CD9 and CD81 and the alpha and beta subunits of integrin were found in cluster 7, which was enriched by *Izon 70 ‐ 3 ml*, *Izon 35*, both *exoEasy* volumes and to some extend *ExoQuick* (Figure 8b). The presence of tetraspanins for the *Izon 70 – 3 ml* was in contrast with the flow cytometry data, were none of the tetraspanins could be detected for these methods. Interestingly, the high presence of EV proteins in the *ExoQuick* samples from plasma but low presence of EV proteins in the conditioned medium samples is in line with the previous data, in which *ExoQuick* did not isolate many EV proteins for the conditioned medium, but isolated the second highest number of EV proteins for the 250 μl plasma samples (Figure 7b). It appears that for conditioned medium, the isolation methods *Izon 70*, *Izon 35 and MagCapture* isolate the more classical EVs (Figure 8a), while for plasma this was true for *Izon 70*, *exoEasy and ExoQuick* (Figure 8b).

The *exoEasy* method, however, also enriches for distinct GO terms which are associated with the endoplasmic reticulum lumen, platelet alpha granule lumen and Golgi lumen (Figure 8a: cluster 1, 8B: cluster 5). Of the proteins included in these clusters, some of them are related to RNA processing, for example, ribosomal proteins RPL19, RPL10A and RPS3A, the RNAses RNASE2 and RNASE4, RNA processing proteins EIF3D and EIF5, and the RNA binding proteins G3BP1 and G3BP2. Interestingly, for the conditioned medium, the *exoEasy* method gave the highest RNA yield (Figure 4d).

The largest cluster in the conditioned medium heatmap (cluster 2) and the second largest cluster in the plasma heatmap (cluster 1) are mainly driven by plasma proteins as multiple lipoprotein members such as ApoA1, ApoA4, ApoC3, ApoE, complement proteins including C3, C5, C6, C7 and C9, and IgG light chains for the plasma cluster were present in these clusters. Thus, these clusters might be considered as a measure of co‐isolated proteins. In accordance with the total number of proteins and number of plasma proteins shown in Figure 7b, for the plasma samples, all methods except *Izon 70 ‐ 250 μl*, contributed to this “non‐EV cluster”. Also, in agreement with earlier results in Figure 7b, for the conditioned medium samples it is mainly *Izon 35, Izon 70* and *exoEasy* that drive this “non‐EV” cluster. Furthermore, although *ExoQuick* showed only a small enrichment for cluster 2, it was nearly absent in all other clusters besides cluster 7, showing that also *ExoQuick* is relatively enriched for cluster 2.

### 14.8 The different isolation methods enrich for diverse vesicles based on cellular compartment classification

**FIGURE 9:**
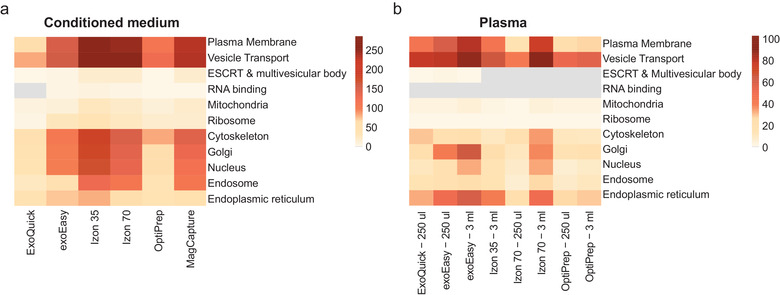
The different methods enrich for distinct EV subpopulations derived from diverse cellular compartments. The identified proteins in each method were categorised for specific cellular compartments. Each cellular component was built with proteins present in selected GO terms corresponding to that specific compartment (supplementary list 4). Next, each of these cellular compartment protein lists were compared with the proteins identified by each isolation method and matching proteins were counted. Results were plotted in a heatmap for the samples from the (a) conditioned medium and (b) plasma. The scale shows the number of counted proteins. If no overlapping proteins were identified, the square was coloured grey for that specific compartment.

After assessing that all of the isolation methods enrich for EVs by using GO annotation, we investigated in more detail whether the methods would isolate different EV types. For this purpose, 11 cellular compartments or machineries were selected, together with their associated GO terms, to find proteins specific for these compartments (Table S4). These 11 compartments or machineries were chosen based on their participation in intracellular vesicle transport (Cytoskeleton and Vesicle Transport), their ability to form intracellular vesicles (Golgi, Endoplasmic Reticulum (ER)), their participation in MV and exosome formation (Plasma membrane and Endosome, ESCRT, and MVBs, respectively), their previous described feature as EV cargo (Mitochondria and Ribosomes) or their measure of unspecific co‐isolation in healthy cells (Nucleus). Subsequently, this list was then used as a criterion to classify the proteins isolated by the different methods of the 11 cellular compartments and cell machineries. Finally, these results were visualized in heatmaps (Figure 9). Important to note is that the classification used here for MVs and exosomes is a very general classification, and to keep in mind that exosomes and MVs can share biogenesis pathways. For example, exosomes can be generated in an ESCRT‐independent way (Trajkovic et al., 2008; Wei et al., 2021), while the ESCRT machinery also has been shown to be involved in MVs generation (Van Niel et al., 2018).

For the conditioned medium samples (Figure 9a), the isolated vesicles from all methods related to the term vesicle transport, confirming the GO annotation data (Figure 8a). Interestingly, for all the conditioned medium samples, though to a lesser extent for *ExoQuick*, the isolated vesicles related to the term plasma membrane, suggesting that all methods enrich for plasma membrane‐derived vesicles, most likely MVs. For the plasma samples, the plasma membrane enrichment was also present for *exoEasy*, both the *Izon* columns and *ExoQuick* (Figure 9b). Moreover, exosomes, as defined by the terms ESCRT & MVB and endosome, were present in the conditioned medium samples isolated by *Izon 35*, *Izon 70* and *MagCapture* and a lesser extent *exoEasy*. In contrast, ESCRT & MVB related proteins were hardly present in the plasma samples based on this analysis. This might be due to very low presence of EVs in plasma as compared to lipoproteins.

## 15 DISCUSSION

The EV field has grown rapidly and its potential is overwhelming, with good prospects both in therapy and clinical diagnostics. To be able to use EVs to their fullest potential it is important to be able to separate them from, or at least identify, their co‐isolates, to understand what type of EV you isolate, and to do so in a time‐ and cost‐effective way. In an attempt to resolve these issues, we have performed a thorough characterisation and comparison of EVs isolated by different isolation methods, from both conditioned MM6 cell medium and human plasma, as they entail different challenges. We performed standard characterisation analyses of the EVs together with an in‐depth proteomic analysis in an attempt to provide insight to which method to use for different samples and applications, and to caution direct comparisons between different studies.

We show that it is of great importance to evaluate the choice of EV isolation method depending on the sample type, volume, and the research question. Especially the sample origin is a significant element in determining which method that is suitable for your study. This is demonstrated by *ExoQuick*, which isolated the least EV‐proteins for the conditioned medium samples, but for the lower plasma volume samples it is the method that isolated the most EV‐proteins. Importantly, this is not only true when comparing fundamentally different sample types such as conditioned cell medium and body fluids, but also for conditioned medium from different cell types or culture conditions, since they will consist of their own specific EV subpopulation diversity (Palviainen et al., 2019; Smith et al., 2015). Notably, we here followed the manufacturers’ instructions when selecting for example fraction numbers, but this should be optimized for each study and sample type.

For conditioned MM6 cell medium, *Izon 35* isolates the highest number of EV proteins, but also co‐isolates the highest amount of non‐EV proteins. However, this method did not isolate significantly more particles, despite the fact that many particles smaller than 30 nm could be detected by EM. Furthermore, it showed the second highest particle‐to‐protein ratio. In contrast, *Izon 70* and *MagCapture* also isolated a high number of EV proteins but with less co‐isolation of non‐EV entities, which might indicate pure EV samples. However, due to the low particle numbers, *MagCapture* also showed the lowest particle‐to‐protein ratio. This demonstrates how important it is not only to evaluate the purity of a sample based on the particle‐to‐protein ratio, but also take in to account what proteins that are actually isolated, as well as the EM results. Moreover, it is important to keep in mind that *MagCapture* will select for a phosphatidylserine‐positive subpopulation of vesicles. In contrast, *exoEasy* yields a lower number of EV‐proteins but the highest protein yield and a significantly higher RNA yield, resulting in a low particle‐to‐protein/RNA ratio. Moreover, it appears that this method biases the isolation of a distinct EV type according to the larger size seen in EM and NTA and the Golgi‐related proteins identified by the proteomic data. Surprisingly, the low particle count in *OptiPrep* still gives a decent total protein yield, resulting in a low particle‐to‐protein ratio. In addition, it isolates a reasonable number of EV‐proteins with low co‐isolation of non‐EV proteins. Lastly, while *ExoQuick* is a straightforward method, it isolates the fewest EV proteins with a co‐enrichment of small particles as seen in EM, presumably LDL, IDL, and VLDL.

When isolating EVs from plasma, these samples show a higher amount of small particles, presumably lipoproteins, and a likely co‐enrichment of free miRNA (Cheng et al., 2020), as seen by the EM, NTA, and Bioanalyzer RNA data. Thus, for plasma the preferred choice of method is a bit different, here *Izon 70* isolates the most EV proteins, but not the purest, as it identified most EV proteins but also the most non‐EV proteins. In addition, we show that although *Izon 70* isolated most EV proteins for plasma, this is only true for larger volumes. For *exoEasy* we show that it enriches for EVs seen by the high number of EV proteins and a lower number of non‐EV proteins detected in the proteomic analysis. However, just as for the conditioned medium samples, this method likely enriches for different vesicle types compared to the other methods, which might bias your results, but might also give opportunities for different biomarker discovery. Interestingly, *ExoQuick* appears to be a better method for plasma samples than for conditioned MM6 cell medium as it, out of the lower plasma volume (250 μl), isolates the most EV‐proteins. Lastly, *Izon 35* and *OptiPrep* isolate the lowest number of EV‐proteins and low amount of RNA but have high presence of tetraspanins as detected by flow cytometry.

Generally, EVs detected by TEM are identified as particles with a cup‐shaped morphology, even though EVs are spherical structures. This cup‐shape is an artefact from TEM sample preparation, caused by sample dehydration (Jung & Mun, 2018; Malenica et al., 2021). Sample preparation can highly affect the structure and visualisation of particles by TEM (Malenica et al., 2021). For example, the different buffers in which the particles are suspended in, can affect the negative stain and make the particles appear more or less dark or have different morphology, leading to different visualisation of the same particles (Scarff et al., 2018). This could be the cause for the dark appearance of the *exoEasy* samples, which has also been identified in other studies (Gutiérrez García et al., 2020; Lipps et al., 2019; Stranska et al., 2018). In addition, not all EVs will present a cup‐shaped morphology in negative stain TEM, making it difficult to distinguish EVs from lipoprotein (Malenica et al., 2021). Negative stain TEM gives a good characterisation of the sample; however, to properly be able to distinguish between EVs and lipoproteins we recommend cryo‐TEM, in which the double membranes of EVs and the single membranes of lipoproteins can be identified, or immunogold negative stain‐TEM in which EVs are labelled for EV enriched markers (Malenica et al., 2021). Lastly, determining the size distribution of your particles using negative stain TEM and NTA both have their drawbacks, as negative stain TEM shrinks vesicles upon dehydration and NTA is not able to accurately detect particles in biological samples smaller than 50 nm (Dragovic et al., 2011). Also, it is not known if NTA overestimates the size of smaller particles close to the detection limit and includes these in the concentration calculation or if these particles are excluded (Bachurski et al., 2019).

Compared to other studies, when either plasma (Karimi et al., 2018; Onódi et al., 2018; Serrano‐Pertierra et al., 2019; Stranska et al., 2018; Takov et al., 2019; Tian et al., 2020), serum (Buschmann et al., 2018; Helwa et al., 2017; Jung et al., 2020; Tang et al., 2017) or conditioned medium (Tang et al., 2017) have been used as a source of EVs, similar particle size and/or concentrations have been shown for both *ExoQuick* (Helwa et al., 2017; Serrano‐Pertierra et al., 2019; Tang et al., 2017) and *exoEasy* (Buschmann et al., 2018; Stranska et al., 2018), showing similar results between our study and previously published studies. In another study by Tian *et al*., (Tian et al., 2020) smaller particle size and a higher concentration of particles among multiple isolation techniques are shown. However, in this study nanoflow cytometry has been used, which provides different results to the more commonly used NTA analysis.

The complexity of plasma has resulted in many studies showing that to achieve a pure EV population, with minimal co‐isolation of lipoproteins and plasma proteins, a combination of several methods are required since they all remove different co‐isolated entities (Brennan et al., 2020; Karimi et al., 2018; Muller et al., 2014; Nordin et al., 2015; Onódi et al., 2018; Tulkens et al., 2020; Vaswani et al., 2017; Zhang et al., 2020). As shown by Karimi *et al*., by combining sequential density gradient and SEC they could remove lipoproteins that are not possible to remove with only one or the other, since these methods are based on density and size, respectively. By first removing the lipoproteins of similar size but of different densities, and then removing lipoproteins that share density with EVs but differ in size, they could achieve the isolation of a purer EV sample, that is, containing a minimal co‐isolation of lipoproteins and plasma proteins (Karimi et al., 2018). These studies showed that for plasma samples it is necessary to apply a combination of methods instead of *OptiPrep* by itself. However, this is only feasible when the sample volume is large enough, as small volumes, such as used in this study, can result in too much loss of EVs when a combination of methods is applied.

Previously, Webber and Clayton hypothesized that pure EV preparations exhibit a relatively high ratio of particles‐to‐protein (Webber & Clayton, 2013). However, we have determined the purity of the EV samples with all results weighing in and not just particle‐to‐protein ratio. This shows that *ExoQuick* does not isolate the purest EV sample, even though it has the highest particle‐to‐protein ratio, since most particles that contribute to the high particle number are smaller than 30 nm and are, therefore, not EVs but rather lipoproteins, since EV are physically unable to be smaller than 30 nm (Huang et al., 2017). Interestingly, System Biosciences now also produces an ExoQuick‐LP kit, containing a lipoprotein pre‐clearing reagent. This might alleviate one of the drawbacks of using a precipitation solution for EV isolation.

Besides assessing EV purity, we also show that the different EV isolation methods isolate different EV subpopulations. Moreover, it has been shown that different EV populations contain different RNA profiles (Crescitelli et al., 2013). This might explain the slightly different RNA profiles between the different EV isolation methods. Importantly, as shown by Crescitelli *et al*., (Crescitelli et al., 2013) EVs derived from different cell lines also have different RNA profiles, thus, the EV RNA profile from conditioned cell medium shown in this study is specific for MM6‐derived EVs. Importantly, also “free” extracellular RNA, for example, RNA associated with Argonaut 2 complexes or ribonucleoprotein complexes, can be co‐isolated together with the EV fractions, which can also play a role in the difference in RNA profiles seen by the different methods (Galvanin et al., 2019; Geekiyanage et al., 2020).

An important finding in the current study is the observed high amount of tetraspanin signal in the “EV‐depleted” fraction 10–12 for *Izon 70*, and to a lower extent in *Izon 35*, indicating that most of our vesicles were present in the flow through fractions. Takov *et al*., (Takov et al., 2017) analysed fraction 7–16 from rat plasma on the presence of CD9, CD81 and APOB using the *Izon 70* column. Their findings are in concordance with ours, as CD9 and CD81 are enriched in fraction 10–12 as opposed to EV enrichment in fraction 7–9 stated by the manufacturer's protocol. In addition, they achieved similar results in a later paper (Takov et al., 2019). Subsequently, since many EVs were found in the flow‐through fraction it is advised that all eluted fractions are carefully analysed before conducting a study using this method, as samples of different origins can have different running and elution times on the SEC column.

Compared to a previous study, we found that EVs isolated by *MagCapture* did not show any CD9 expression as shown by flow cytometry, while it was previously shown that these vesicles do express CD9 (Nakai et al., 2016). However, CD9 was detected in one of the conditioned MM6 cell medium samples by LC‐MS/MS, but in a ∼ 5 times lower amount compared to other methods, so this is rather a detection level effect. In addition, the difference in CD9 expression between our study and others could be due to different cell sources used. Important to keep in mind is that, although phosphatidylserine has been shown to be expressed in the outer leaflet of cancer‐derived and apoptotic cell‐derived EVs, there is still no clear consensus whether this lipid is normally present in the outer leaflet of exosomes (Skotland et al., 2017).

When analysing the proteomic data, we showed that proteins normally not considered as EV proteins such as Golgi, ribosomal and endoplasmic reticulum derived proteins were enriched by *exoEasy*, *Izon 35*, *Izon 70* and *MagCapture* for the conditioned medium samples. For the plasma samples, Golgi related proteins were detected in the *exoEasy* and *Izon 70*, while ER‐related proteins were detected in *exoEasy*, *Izon 35*, I*zon 70* and to some extent *ExoQuick*. Interestingly, the data from the cellular compartment classification together with the GO enrichment data, indicates that these non‐EV proteins might not be actually co‐isolated proteins as previously thought, but rather proteins that are specific to certain subtypes of EVs. Indeed, Golgi‐related proteins have been identified in low density small EVs (Crescitelli et al., 2020). Moreover, proteins related to ribosome and mitochondria have been previously identified in mainly larger EVs (Jimenez et al., 2019; Kowal et al., 2016), which could also be detected in our samples for both conditioned cell medium and plasma. Also it was shown for small EVs (100 K pellet) that they were, besides MVB and endosome related proteins, enriched for adhesion molecules (Jimenez et al., 2019). This is in line with our data which showed that all methods, to a lesser extent for *OptiPrep* and *ExoQuick*, were enriched for the GO term focal adhesion. Recently, also ER‐ associated intracellular vesicles have been described (Carter et al., 2020), although whether these can be released from cells is not clear. It would be interesting to determine if *Izon 35* and *exoEasy*, which had the highest presence of ribosome associated proteins, might enrich for these particles.

Besides the quality and quantity of the EVs isolated by each method, there are practical things to consider such as the number of samples, cost, (hands on) time, specialised equipment and user difficulty (Table 5). Additionally, an isolation method may require training and results can be subject to interpersonal differences of isolation, creating low reproducibility. This is clearly illustrated by our data where *exoEasy* and *ExoQuick* have a similar expression pattern between the samples, while *OptiPrep* and *Izon 70* haver more inter‐sample differences (Figure 8). In general, *OptiPrep* and the *Izon* columns have much more hands on time, which increases the risk of human error. Furthermore, they are very time consuming and for *OptiPrep* expensive equipment, that is, an ultracentrifuge is also required; therefore, these methods might not be the optimal methods of EV isolation from a large number of plasma samples in a biomarker study, especially if the sample volume is low. In contrast, *exoEasy* and *ExoQuick* are fast methods and are easier to perform, so these methods might be a better alternative when big sample groups or larger sample amounts need to be processed. However, *exoEasy* is the most expensive method, at least in Europe in the year of 2021.

**TABLE 5:** Practical information about the different EV isolation methods

Isolation method	Cost	Total time conditioned medium/plasma (per one isolation)	Hands on time	Maximum load (conditioned medium/plasma)	Specialized equipment	Easy to perform
ExoQuick ULTRA	€€€	15.5 h / 1.5 h	45 min	250 μl	No	++
exoEasy	€€€	30 min	30 min	16 ml / 4 ml	No	+++
Izon 35	€	3–4 h	3–4 h	500 μl	No	+
Izon 70	€	1.5–2 h	1.5–2 h	500 μl	No	+
OptiPrep	€	19 h	2 h	N/A	Yes^a^	+
MagCapture	€€	4 h	1h	1 ml	No	++

Time and price is based on one isolation using the maximum isolation volume. Prices are based on items purchased in Sweden 2020.

Cost: € = ≤10 €/isolation, €€ = 11–25 €/isolation, €€€ = > 25 €/isolation; Easy to perform: +++ = easy, ++ = medium, + = hard.

^a^ Ultracentrifuge.

In conclusion, different methods for EV isolation yield different products and the choice depends on the clinical or scientific application. Importantly, our results also highlight the importance of carefully evaluating the EV isolation method when comparing the results in published studies using different isolation methods.

## 16 CODE AVAILABILITY AND PROTEIN LISTS

Codes used in this paper are available on Github (https://github.com/RoosVeerman/ev_isolation). Furthermore, lists of proteins identified by each method are also stored there. Moreover, lists of the identified proteins of the Venn diagrams in Figure 7, and lists of the protein present in each cluster in Figure 8 can also be found there.

## CONFLICT OF INTEREST

Susanne Gabrielsson has a patent on B cell derived exosomes in immune therapy and is part of the Scientific Advisory Board of Anjarium Biosciences.

## AUTHOR CONTRIBUTION

Conception and design: Rosanne E. Veerman, Gözde Güclüler Akpinar, Susanne Gabrielsson, Maria Eldh. Development of methodology: Rosanne E. Veerman, Loes Teeuwen, Maria Eldh. Acquisition of data: Rosanne E. Veerman, Loes Teeuwen, Xiaofang Cao, Maria Eldh. Analysis and interpretation of data: Rosanne E. Veerman, Loes Teeuwen, Paulo Czarnewski, Gözde Güclüler Akpinar, AnnSofi Sandberg, Xiaofang Cao, Maria Pernemalm, Lukas M. Orre, Susanne Gabrielsson, Maria Eldh. Writing outline of manuscript: Rosanne E. Veerman, Loes Teeuwen, Maria Eldh. Reading and revision of manuscript: all authors.

## Supporting information

Supplementary Figure 1. Morphological analysis by negative stain transmission electron microscopy (TEM) of particles present in the non‐EV fractions using *Izon 35*, *Izon 70* and *OptiPrep* from (A) conditioned medium from MM6 cells and (B) human plasma. (C) “EV‐enriched” fractions 7–9 of serum free conditioned cell medium and non‐conditioned EV‐depleted culture medium using *Izon 70*. Last image is non‐conditioned EV‐depleted culture medium without concentration and isolation. Scale bars are 1 μm except, where indicated 500 nm. High Density (HD), Low Density (LD).

Supplementary Figure 2: Bovine derived miR‐122 is absent in all samples isolated by the different methods. (A) RT‐PCR of miR‐122 in samples containing FBS‐derived EVs, MM6‐derived EVs, both isolated by ultracentrifugation, and MM6‐cells cultured in presence of 10% EV depleted FBS or in the absence of FBS. The MM6‐derived EVs cultured with EV depleted FBS still have some miR‐122 present, showing that ultracentrifugation does not completely deplete FBS of miR‐122. miR‐122 was absent in both EVs and cells cultured with EV depleted FBS, as seen by Cq above 35. (B) RT‐PCR of miR‐122 of conditioned medium samples isolated by ultracentrifugation (blue), ExoQuick, exoEasy, Izon 35, Izon 70, MagCapture. miR‐122 was equal in EVs isolated by all methods as Quantification cycle (Cq) were similar. Not detected (N.D.)

Supplementary Figure 3: FACS flow through (Izon 70 & Optiprep). EVs from both the EV fractions and the non‐EV fraction (flow‐through fractions for Izon 35 and Izon 70; high density fraction and low‐density fraction for OptiPrep) were bound to anti‐CD9 or anti‐CD63 coated latex beads and flow cytometry was used to show the presence of the surface markers CD9 (blue), CD63 (grey) and CD81 (purple). (A) Conditioned medium and (B) plasma derived samples. Data is shown as MFI ratio between specific antibody and the corresponding isotype control. A signal above 1 (dotted line) was considered as a positive signal. Data are represented as mean values and SD, *n* = 3. Data analysed by one‐way ANOVA with Tukey's HSD for multiple comparisons for each marker (CD9, CD63, CD81), *P < 0.05, **P < 0.01, and ***P < 0.001.

Supplementary Figure 4: PCA shows no batch effect and separate clustering of the plasma and conditioned medium samples. (A) No batch effect was found for the 2 additional MM6 cell culture batches. Principal Component Analysis (PCA) was performed on all proteins identified by mass spectrometry in the conditioned medium samples. New batches of MM6 conditioned medium were prepared for *Izon 35*. For exoEasy, the two later added MM6 cell culture batches (batch 3 and 4) clustered together with the previously prepared batches for the exoEasy method. Therefore, it was concluded that the separation of *Izon 35*, which was isolated from batch 3 and 4, is a true separation and not based on batch effect. (B) PCA was performed on all samples, both plasma and conditioned medium. Clear separation of the sample types in PC1 was observed.

Supplementary information

Supplementary information

Supplementary information

Supplementary information
