# Supplementary figures and images for: Molecular evaluation of five different isolation methods for extracellular vesicles reveals different clinical applicability and subcellular origin
Source: J Extracell Vesicles. 2021 Jul 22;10(9):e12128. doi: 10.1002/jev2.12128 (PMC8298890; doi:10.1002/jev2.12128)

Supplementary figure 1

A

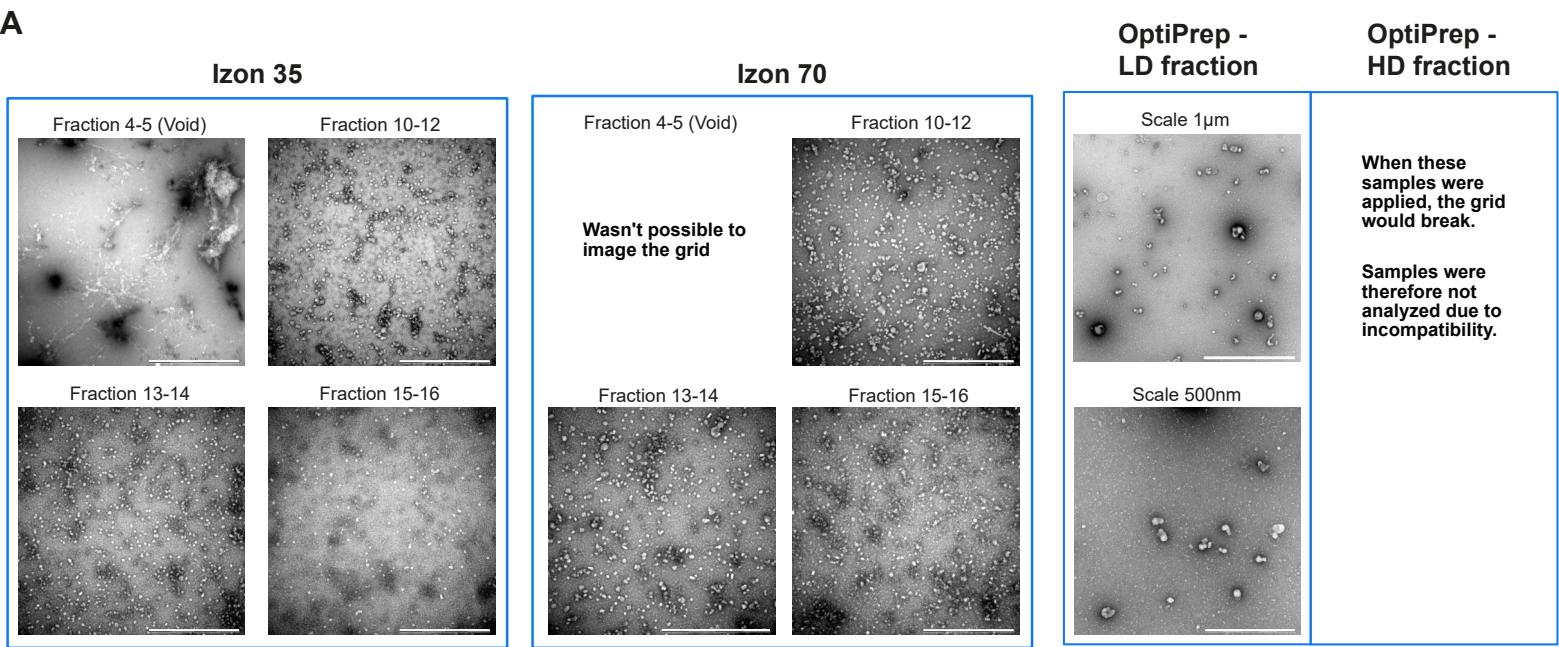

B

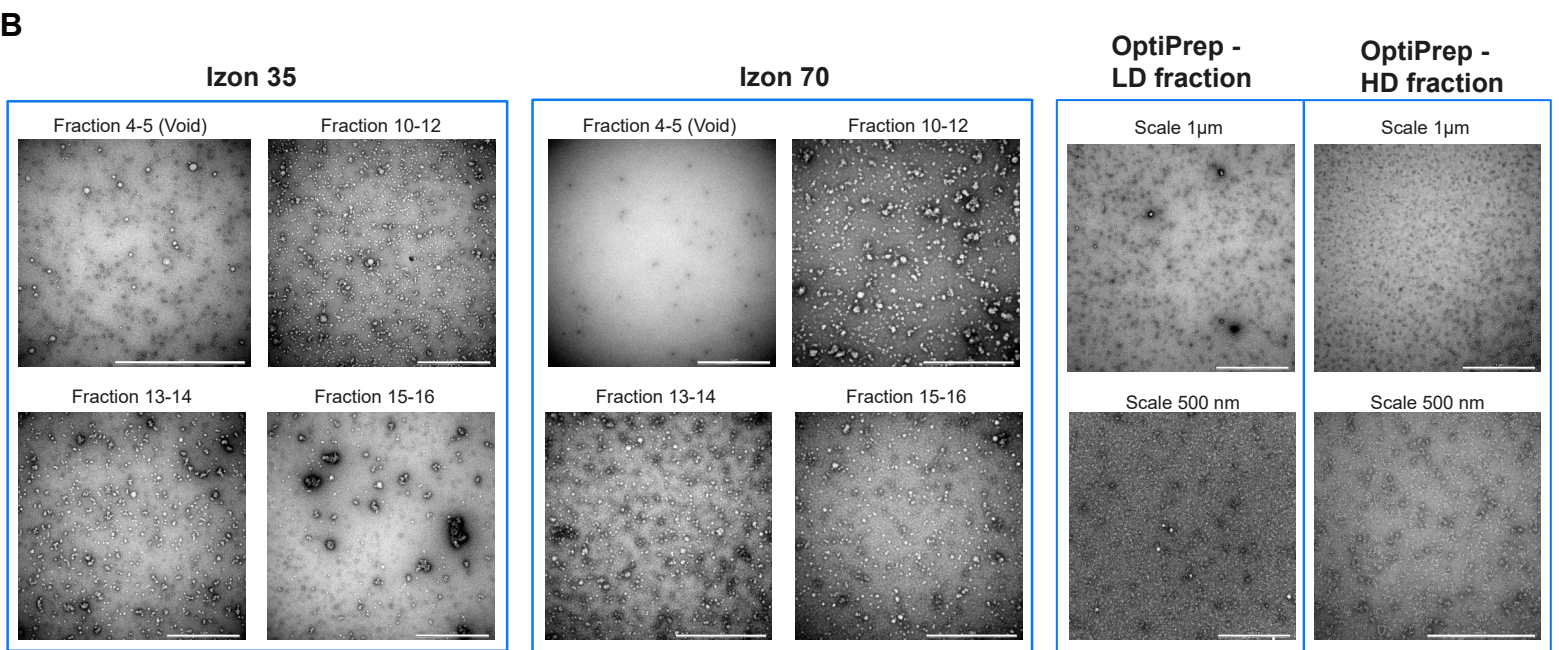

C

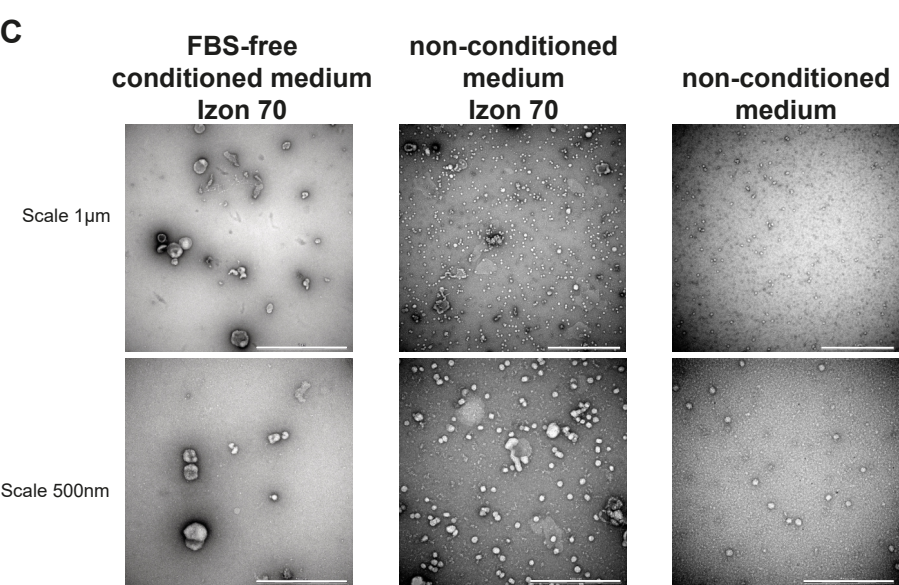

Supplement: Supplementary file 1 — Supplementary Figure 1. Morphological analysis by negative stain transmission electron microscopy (TEM) of particles present in the non‐EV fractions using Izon 35, Izon 70 and OptiPrep from (A) conditioned medium from MM6 cells and (B) human plasma. (C) “EV‐enriched” fractions 7–9 of serum free conditioned cell medium and non‐conditioned EV‐depleted culture medium using Izon 70. Last image is non‐conditioned EV‐depleted culture medium without concentration and isolation. Scale bars are 1 μm except, where indicated 500 nm. High Density (HD), Low Density (LD). [file JEV2-10-e12128-s008.pdf]

Supplementary figure 2

A

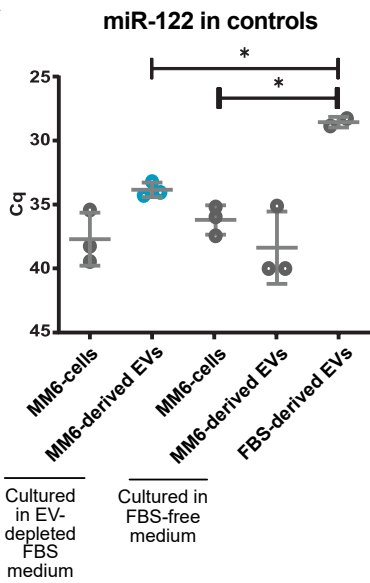

B

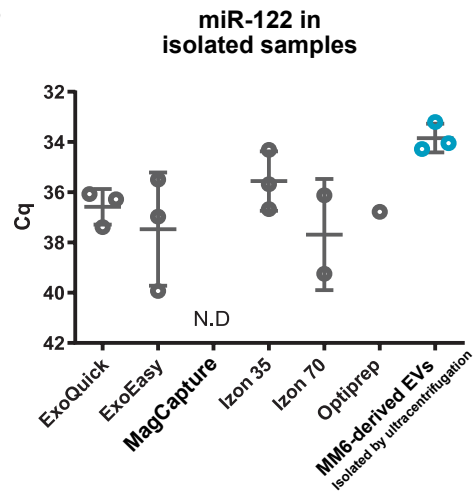

Supplement: Supplementary file 2 — Supplementary Figure 2: Bovine derived miR‐122 is absent in all samples isolated by the different methods. (A) RT‐PCR of miR‐122 in samples containing FBS‐derived EVs, MM6‐derived EVs, both isolated by ultracentrifugation, and MM6‐cells cultured in presence of 10% EV depleted FBS or in the absence of FBS. The MM6‐derived EVs cultured with EV depleted FBS still have some miR‐122 present, showing that ultracentrifugation does not completely deplete FBS of miR‐122. miR‐122 was absent in both EVs and cells cultured with EV depleted FBS, as seen by Cq above 35. (B) RT‐PCR of miR‐122 of conditioned medium samples isolated by ultracentrifugation (blue), ExoQuick, exoEasy, Izon 35, Izon 70, MagCapture. miR‐122 was equal in EVs isolated by all methods as Quantification cycle (Cq) were similar. Not detected (N.D.) [file JEV2-10-e12128-s007.pdf]

# Supplementary figure 3

A

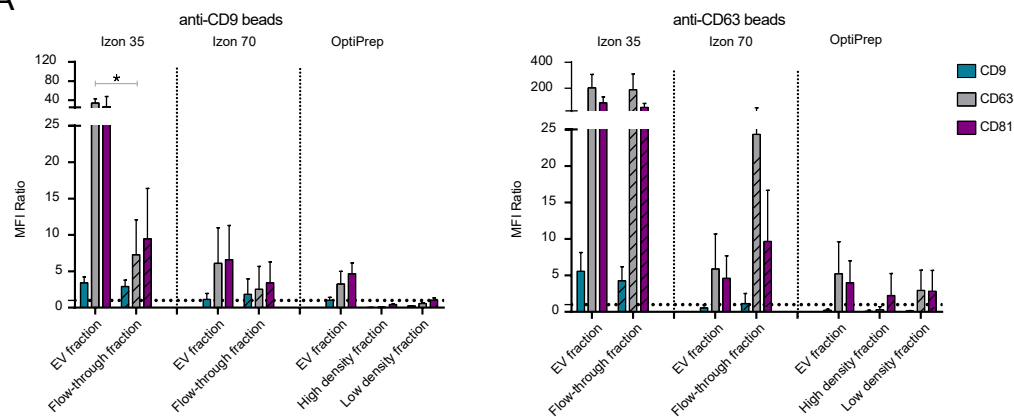

B

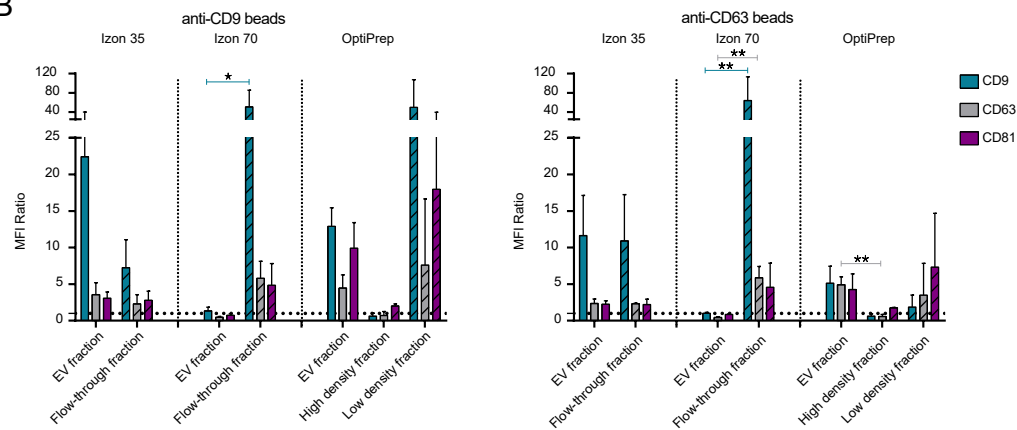

Supplement: Supplementary file 3 — Supplementary Figure 3: FACS flow through (Izon 70 & Optiprep). EVs from both the EV fractions and the non‐EV fraction (flow‐through fractions for Izon 35 and Izon 70; high density fraction and low‐density fraction for OptiPrep) were bound to anti‐CD9 or anti‐CD63 coated latex beads and flow cytometry was used to show the presence of the surface markers CD9 (blue), CD63 (grey) and CD81 (purple). (A) Conditioned medium and (B) plasma derived samples. Data is shown as MFI ratio between specific antibody and the corresponding isotype control. A signal above 1 (dotted line) was considered as a positive signal. Data are represented as mean values and SD, n = 3. Data analysed by one‐way ANOVA with Tukey's HSD for multiple comparisons for each marker (CD9, CD63, CD81), *P < 0.05, **P < 0.01, and ***P < 0.001. [file JEV2-10-e12128-s006.pdf]

# Supplementary figure 4

A

## Conditioned medium

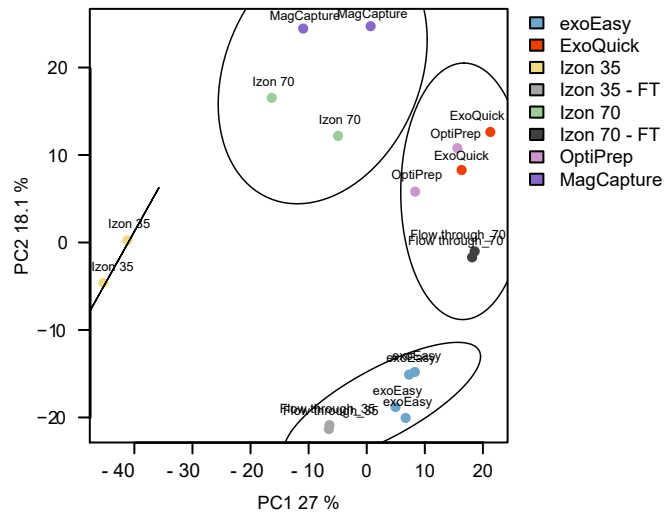

B

## All samples

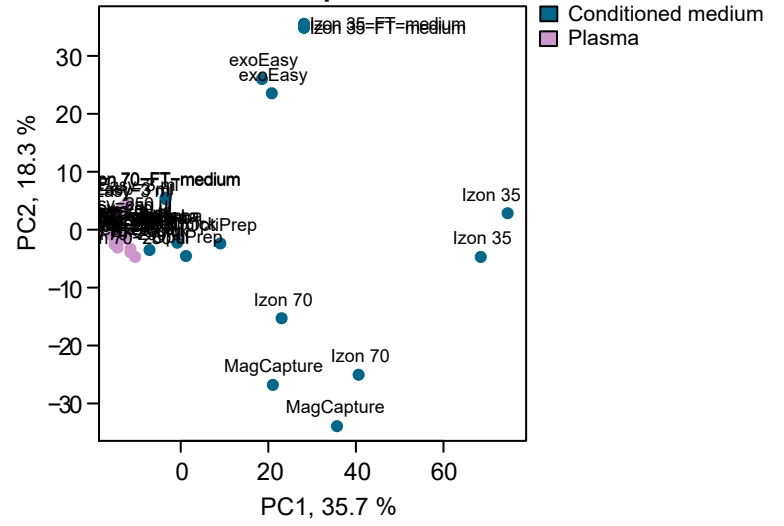

Supplement: Supplementary file 4 — Supplementary Figure 4: PCA shows no batch effect and separate clustering of the plasma and conditioned medium samples. (A) No batch effect was found for the 2 additional MM6 cell culture batches. Principal Component Analysis (PCA) was performed on all proteins identified by mass spectrometry in the conditioned medium samples. New batches of MM6 conditioned medium were prepared for Izon 35. For exoEasy, the two later added MM6 cell culture batches (batch 3 and 4) clustered together with the previously prepared batches for the exoEasy method. Therefore, it was concluded that the separation of Izon 35, which was isolated from batch 3 and 4, is a true separation and not based on batch effect. (B) PCA was performed on all samples, both plasma and conditioned medium. Clear separation of the sample types in PC1 was observed. [file JEV2-10-e12128-s005.pdf]
